# Emergent Insulator–Metal Transition with Tunable Optical and Electrical Gap in Thin Films of a Molecular Conducting Composite

**DOI:** 10.1021/acsaelm.2c00224

**Published:** 2022-05-11

**Authors:** Raphael Pfattner, Elena Laukhina, Jinghai Li, Rossella L. Zaffino, Núria Aliaga-Alcalde, Marta Mas-Torrent, Vladimir Laukhin, Jaume Veciana

**Affiliations:** † Institut de Ciència de Materials de Barcelona (ICMAB-CSIC), Campus UAB, 08193 Bellaterra, Spain; ‡ Networking Research Center on Bioengineering Biomaterials and Nanomedicine (CIBER-BBN), Campus UAB, 08193 Bellaterra, Spain; § ICREA−Institució Catalana de Recerca i Estudis Avançats, Passeig Lluis Companys 23, 08010 Barcelona, Spain

**Keywords:** molecular metal, composites, insulator−metal transition, tunable bandgap, optoelectronic properties

## Abstract

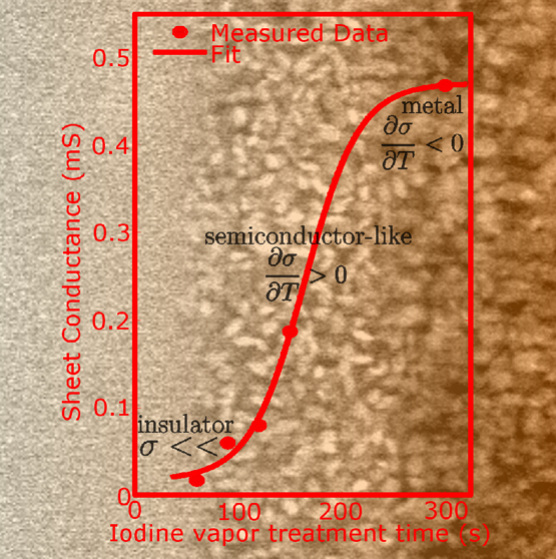

Composites exhibit unique synergistic properties emerging when components with different properties are combined. The tuning of the energy bandgap in the electronic structure of the material allows designing tailor-made systems with desirable mechanical, electrical, optical, and/or thermal properties. Here, we study an emergent insulator–metal transition at room temperature in bilayered (BL) thin-films comprised of polycarbonate/molecular-metal composites. Temperature-dependent resistance measurements allow monitoring of the electrical bandgap, which is in agreement with the optical bandgap extracted by optical absorption spectroscopy. The semiconductor-like properties of BL films, made with bis(ethylenedithio)-tetrathiafulvalene (BEDT-TTF or ET) α-ET_2_I_3_ (nano)microcrystals as two-dimensional molecular conductor on one side and insulator polycarbonate as a second ingredient, are attributed to an emergent phenomenon equivalent to the transition from an insulator to a metal. This made it possible to obtain semiconducting BL films with tunable electrical/optical bandgaps ranging from 0 to 2.9 eV. A remarkable aspect is the similarity close to room temperature of the thermal and mechanical properties of both composite components, making these materials ideal candidates to fabricate flexible and soft sensors for stress, pressure, and temperature aiming at applications in wearable human health care and bioelectronics.

## 1 Introduction

For the past two centuries, technological advances have emerged side by side with the development of novel materials with tunable electrical, optical, and/or mechanical properties. About the latter, the most searched have been materials showing good electrical and thermal conductivities, using these criteria to classify them. This way, classical metals do exhibit high conductivity, due to the absence of an energy gap in their electronic structure and the high number of free electrons moving in a gaslike fashion. Their optical properties are closely related to this, and metals are typically shiny and not transparent to light. Semiconductors, on the other hand, enable tunable optoelectronic properties due to a forbidden energy gap. The value of this bandgap can make them transparent to electromagnetic radiation of different wavelengths but active (the material absorbs) at others, opening up a variety of applications.^1^ This definition is not limited to materials consisting of single atoms, and many molecule-based materials can exhibit similar or even greater tuneability of their optoelectronic properties.^2^

As a paradigmatic example, one can mention crystals of bis(ethylenedithio)-tetrathiafulvalene (BEDT-TTF or ET) salts with halogen-based counterions that show semiconducting, metallic, or even superconducting properties,^3^ where the anisotropic electronic composition, caused by lattice deformations, promotes fascinating electronic and structural phase transitions that can be controlled by external stimuli such as light, temperature, strain, pressure, and humidity, bringing the opportunity to use them as sensors of such stimuli.^4−8^ When engineered into a proper material for applications, by forming polycrystalline layers of ET salts in nanocomposite bilayer (BL) films, the fascinating electrical properties of these single crystals are combined with classical properties of insulating polymers, like lightness, flexibility, and manufacturing ability such as pattern ability, mold ability, and process ability.^9−11^ Similarly, such BL films have the additive value of exhibiting novel synergistic, greater than the sum, properties appearing when the different components combine.^12^ Aforesaid outcomes are similar to polymer-based nanocomposites with other conductive fillers (*i.e*., carbon black, carbon nanotubes, graphene flakes, *etc.*) which have been extensively explored due to their high-function tuneability.^13−15^

Nanocomposite BL films of ET salts with I_3_^–^ anions are made by exposing a casted thin-film comprised of a solid solution of neutral ET molecules in a polymer, like poly(bisphenol A-carbonate (PC)) to an iodine vapor together with vapors of an organic solvent like dichloromethane (DCM) during a period for more than 300 s. In this way, a well-developed network of intertwined ET_2_I_3_ (nano)microsized crystallites is formed on top of the film. By controlling the temperature during iodine treatment, it is possible to manage the formation of different crystalline polymorphs of ET_2_I_3_ salts, *i.e.*, the α- or β-phases, and thereby attaining a fine-tuning of the resistance temperature dependence, resulting in BL films with either a semimetal or a metallic behavior.^16^

Previous works showed the influence of temperature during the BL formation by iodine treatment but lacked in displaying information on the generation and evolution of crystallites. In the current work, we study the time evolution of growing crystallites in the BL film formation while keeping a constant temperature during the halogen treatment. This allows the monitoring of the nucleation and evolution of crystallites and, at the same time, the formation of BL films with different evolving conducting properties: starting from the insulating property of the pristine PC, followed by a semiconductor-like behavior in an intermediate step, and finally attaining a metallic performance at high treatment times, where the generated composite conductivity values approach those reported for single crystals of the molecular metal. Here, the latter insulator–metal transition has been successfully studied and described by the percolation theory. Furthermore, the electrical bandgap of BL films obtained at different treatment times has been monitored by temperature-dependent resistance measurements, while the optical bandgap was studied by optical absorption spectroscopy resulting in similar values for both. Also, the chemical composition and morphology of the conducting layer has been analyzed by scanning electron microscopy (SEM) images in combination with energy-dispersive X-ray (EDX) spectroscopy. Our results show that the semiconductor-like property of BL films containing α-ET_2_I_3_ crystals in the intermediate step is attributed to an emergent phenomenon in the transition from an insulator (PC) to the two-dimensional molecular metal α-ET_2_I_3_ (*vide infra*). Our findings broaden the scope of novel composites with tailor-made optoelectronic properties and with useful practical applications. Indeed, by wisely selecting polymer matrixes and conductive molecule-based crystals as well as controlling the preparation conditions (*i.e.*, time and temperature), new materials with desirable mechanical, electrical, optical, and thermal properties can be designed, suitable for state-of-the-art applications targeting remote medicine^17^ and human health care.^18,19^

## 2 Results and Discussion

### 2.1 Chemical Composition, Morphology, and Structure of Topmost Layer in BL Films

**Figure 1:**
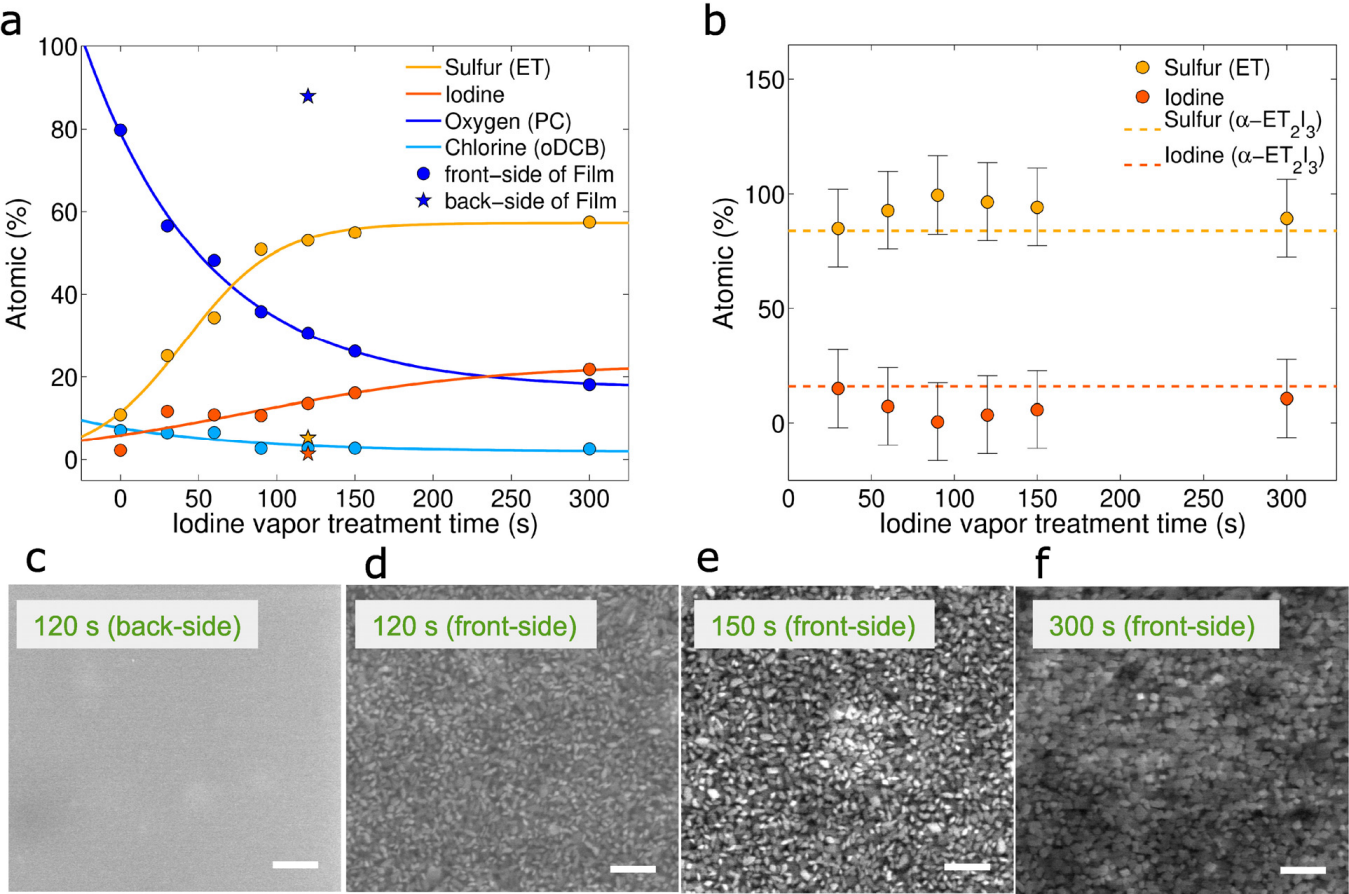
Chemical composition and scanning electron microscopy images of BL films. (a) Chemical composition of BL films in atomic weight percent (atomic %) extracted employing EDX analysis as a function of iodine vapor treatment time. Continuous lines for oxygen and chlorine are fitted to an exponential decay, while sulfur and iodine signals are fitted to a Boltzmann sigmoid. (b) Sulfur/iodine atomic concentration with the estimated theoretical stoichiometry for α-ET_2_I_3_ shown as broken lines. SEM images were taken in the low vacuum secondary electron (LFD) mode of the (c) backside of the film and (d–f) front side for halogen vapor treatment times of 120 s, 150 s, and 300 s, respectively. Scale bar corresponds to 2 μm.

SEM images in combination with EDX are powerful tools to extract the morphology and the atomic composition of materials. Figure 1 shows the elemental analysis of BL films treated with increasing iodine vapor treatment times at *T* = 25 ± 1 °C. The pristine sample (*i.e.*, at time *t* = 0 s, before exposure to halogen vapors) exhibited mostly oxygen (O = 79.8%, corresponding to polycarbonate), sulfur (S = 10.9%, related to neutral ET), followed by low concentrations of chlorine atoms (Cl = 7.1%, consistent with the presence of *ortho*-dichlorobenzene (*o*DCB), the solvent for the film casting. The very low concentration of iodine (I = 2.2%), not present at *t* = 0 s, was used to assess the sensitivity of the elemental analysis. Upon increasing the halogen vapor treatment times, oxygen (from PC) and chlorine (from *o*DCB and dichloromethane, DCM, solvents)^20^ amounts decrease following an exponential decay with time constants of τ_O_ = 78 ± 16 s and τ_Cl_ = 90 ± 100 s, respectively [At(*t*) = At_*t*_0__ exp(−*t*/τ_At_) + At_*t*_∞__]. The high error extracted for the chlorine assessment relates to the fact that there is a very little amount of processing solvent left in the samples that further decreases with halogen vapor treatment time. The level of trapped solvent is particularly important when aiming at biomedical applications and the biocompatibility of BL films, as previously explored in a prototype to monitor the intraocular pressure.^19^ Both sulfur and iodine content, on the other side, increases according to a Boltzmann sigmoid, [At(*t*) = At_*t*_0__ + (At_*t*_∞__ – At_*t*_0__)/(1 + exp[(*t* – *t*_m_)/τ_At_])], with typical time constants of τ_S_ = 29 ± 7 s and τ_I_ = 78 ± 45 s, respectively. The high error found in this case for the iodine signal relates to the resolution of this technique. Estimated values of the fully grown and phase-separated polycrystalline layer, which occurred at treatment times larger than 300 s, were found to be S_*t*_∞__= 57 ± 6%, I_*t*_∞__ = 23 ± 25%, and O_*t*_∞__ = 17 ± 6%. The high oxygen level is most probably attributed to the penetration depth of the EDX analysis which reaches the PC beneath the topmost polycrystalline layer. Additionally, a sample treated for *t* = 120 s was mounted with the topmost conducting layer facing downward, thus exposing the back of the sample (shown in Figure 1a, star symbols) and exhibited mostly oxygen corresponding to PC. All the above-described facts indicate a progressive diffusion of the neutral donor molecule (ET) toward the surface, favored by the polymer swelling induced by vapors of DCM that reacts with iodine forming a topmost, phase-separated polycrystalline layer. As it will be shown later, the above-reported time constants from EDX measurements agree well with exponential decay constants extracted in the electrothermal characterizations, showing a clear correlation. Figure 1b depicts the sulfur–iodine atomic concentration extracted from data shown in Figure 1a, which allows estimating the sulfur/iodine ratio. Within the measurement resolution of the technique, all values of atomic composition agree with the theoretical stoichiometry for an S/I ratio of 84.2/15.8 expected for a single crystal of α-ET_2_I_3_, shown with dashed lines in Figure 1b. Following our analysis, Figure 1c exhibits a SEM image of the backside of the BL films, while Figure 1d–f shows the topmost polycrystalline layer measured at different treatment times where a continuous growing layer of nano(micro)crystallites comprising grains and boundaries is visible (see also full images and additional info on EDX analysis, Supporting Information sections S1 and S4).

In order to study the chemical composition perpendicular to the BL film surface, a sample treated for *t* = 300 s was cut and mounted in the vertical position. The EDX scan along the cross section revealed high concentrations of both sulfur and iodine adjacent to the front side of the sample. This region corresponds to an α-ET_2_I_3_ polycrystalline layer, and its thickness was estimated to be about *d*_α-ET2I3_ = 1.48 ± 0.02 μm (please see Supporting Information section S2 for more details on the cross section analysis).

### 2.2 Percolation Theory to Study Composites: BL-Films

**Figure 2:**
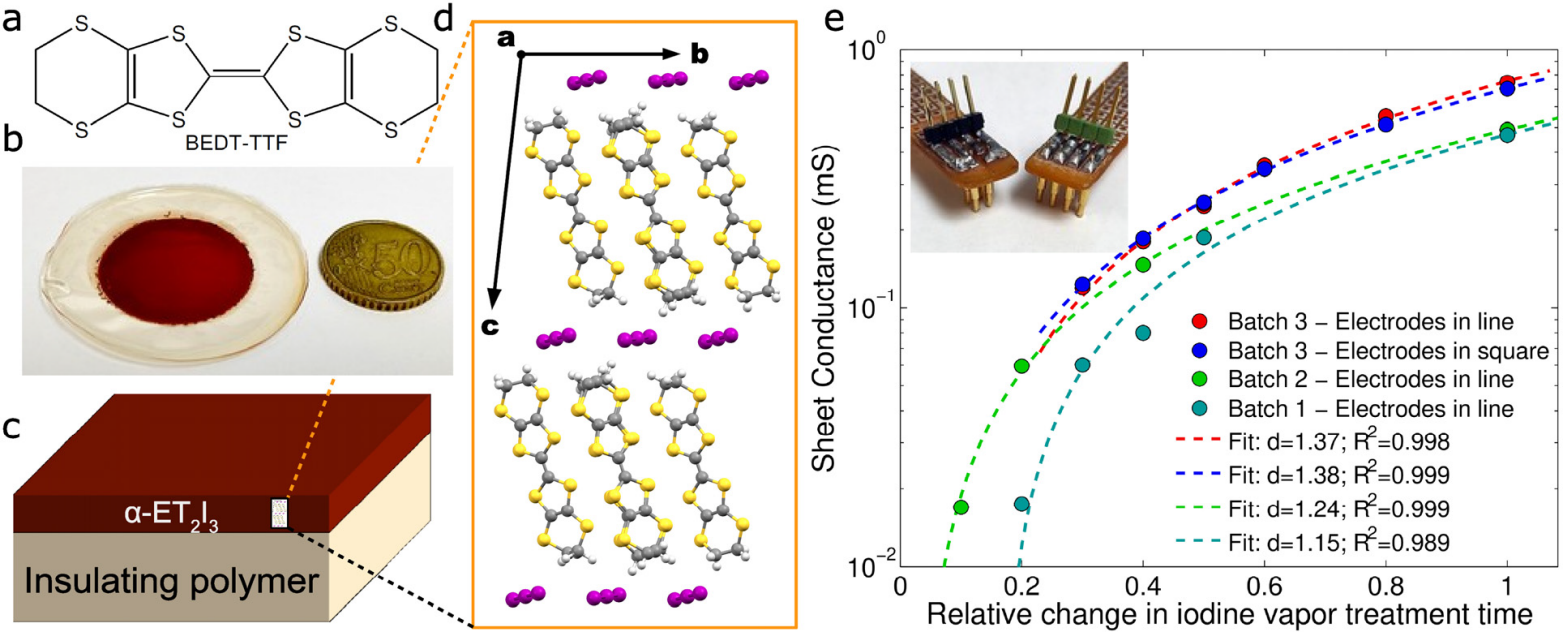
Structure and electrical characteristics for nanocomposite bilayer films. (a) Chemical structure of bis(ethylenedithio)tetrathiafulvalene (BEDT-TTF or ET), (b) optical image of BL film treated with iodine vapors, (c) schematic representation of BL films comprising polycarbonate (PC) matrix with a topmost polycrystalline network of α-ET_2_I_3_ (see Supporting Information for the thicknesses of both layers). (d) View of the crystal packing of the salt with the crystal axis (**a**) perpendicular to the text. (e) Sheet conductance of a few batches of films prepared with increasing relative iodine vapor treatment times (*i.e*., *p* = time/time_300s_) and measured with different electrode geometries (see inset). Dashed lines represent best fits according to the percolation theory.

Figure 2 shows the molecular structure of a neutral ET molecule along with an optical image of a typical BL film of crystallites made on a glass support. As reported in an earlier publication,^6^ the fabrication process of such BL films, obtained for longer iodine vapor treatment times (>300 s), leads to a continuous topmost layer of intertwined and oriented α-ET_2_I_3_ crystallites, ascertained by X-ray diffraction techniques.^6^ Such crystallites have a preferred orientation with their crystallographic (c* = **a × b**) direction almost perpendicular to the BL surface film, whereas their (**a**–**b**) planes are randomly oriented parallel to the BL film surface. Thus, the neutral ET and oxidized ET^+^ molecules of α-ET_2_I_3_ crystallites are orientated almost in an upright position and sandwiched between the I_3_^–^ anion layers, with the π–π stacking direction of ET molecules along the (**a**) axis and the π–π interstacking along the (**b**) one, respectively. A view of the crystal structure of α-ET_2_I_3_, seen along the crystallographic axis (**a**), is depicted in the enlarged view (orange rectangle), Figure 2d.^21,22^ Single crystals of α-ET_2_I_3_ exhibit a high electrical anisotropy with a quasi-two-dimensional metallic behavior due to the molecular stacking.^21^ Indeed, they show the highest electrical conductivity when measured along the π–π interstacking direction of ET molecules (*i.e*., the **b** axis) with σ_||,b_ = 200 (S/cm), due to short intermolecular S–S contacts, while a slightly lower value of about σ_||,a_ = 100 (S/cm), was found along the crystal (**a**) axis, due to π–π interactions.^21^ However, perpendicular to the (**a**–**b**) plane (*i.e*., at direction c* = **a × b**), the crystal shows the lowest conductivity with σ_⊥,c*_ = 0.2 (S/cm), due to the presence of insulating I_3_^–^ anion layers. The network of micro/nanocrystals in BL films reproduce the same high electrical anisotropy as the single crystals, since isotropic electrical characteristics were observed in the plane (*vide supra*) with a value of about σ_line_ = 1.35 ± 0.05 S/cm and σ_square_= 1.32 ± 0.05 S/cm.^23^ These values are small when we compare with those of σ_||,b_ and σ_||,a_ but much higher than the σ_⊥,c*_ value observed in single crystals. This result is related to the fact that the crystallites are electrically connected through their most conducting edge with boundaries limiting the overall conductivity of the BL film, confirming thus the quasi two-dimensional conductivity of the network of micro/nanocrystals in BL films.

In order to monitor the growing of crystallites on the top surface during the BL formation, we followed the resulting conductance at different iodine vapor processing times. Figure 2e shows the sheet conductance of the polycrystalline layer measured at different courses of time by assuming a zero percolation at time = 0 s (*i.e*., a null formation probability of a continuous topmost polycrystalline layer, *p* = 0) and a total probability of a continuous polycrystalline layer formation at higher treatment times >300 s (*i.e*., *p* = 1). According to classical percolation theory, an insulator–conductor transition takes place when the volume fraction of the conductive filler approaches the percolation threshold. At low loading values, the conductivity of such electronic systems is often characterized, using a percolation power-law dependency in the form of σ = σ_0_ (ϕ – ϕ_c_)^*d*^,^24^ where σ_0_ is a constant of proportionality (the upper limit for maximum conductivity at 100% loading), ϕ is the volume fraction of the conducting filler in the composite, ϕ_c_ is the critical volume fraction above which percolation can occur, and *d* is the transport exponent accounting for the dimensionality of the charge transport. For the studied BL films, the volume fraction at each iodine treatment time is not directly accessible, but taking into account that the crystallites formation on the surface of the film is a diffusion-limited process,^25^ a direct relationship between the normalized treatment time and the probability to find crystallite on the surface must be expected (*i.e*., *p* = α-ET_2_I_3_/(α-ET_2_I_3_ + PC); ratio ≈ time/time_300s_, see also Supporting Information section S3). According to that, the conductance of the topmost growing α-ET_2_I_3_ micro/nanocrystals follows a power law dependency like σ = σ_0_ (*p* – *p*_c_)^*d*^, where *p* is the formation probability of the topmost polycrystalline layer, *p*_c_ the percolation threshold, and *d* relates again to the critical transport exponent. This is plausible, since the universality principle, developed for percolation theory, states that the numerical value of *p*_c_ is determined by the local structure of the material, whereas the critical exponent (*d*) does not depend on the microscopic details of the lattice but only on the Euclidean dimension of the conducting fillers.^26,27^ In other words, this means that before the halogen vapor treatment, the electrical property of the composite is limited to the bulk conductivity of the insulating matrix, leading to an insulating response. With the increasing concentration of conducting fillers (*i.e*., growing crystallites on the surface), the charge transport increases in accordance with the critical exponent. Theoretical simulations for materials with percolation pathways exhibit critical exponents of *d* = 1.30 ± 0.01 and *d* = 2.26 ± 0.04 for 2D and 3D systems, respectively.^28,29^ The critical exponent extracted from the measurements of three different batches of α-ET_2_I_3_-based BL films, employing two different electrode geometries (*vide infra*), leads to a mean value of about *d* = 1.28 ± 0.11, in good agreement with the theoretical value expected in the presence of 2D metallic conducting fillers, as it is the case of the quasi-2D metallic α-ET_2_I_3_ crystallites. The extracted upper limit of the composites sheet conductance was fitted to a value of σ_0_ = 0.66 ± 0.15 mS, with a corresponding percolation threshold of *p*_c_ = 0.07 ± 0.06. This provides a critical percolation treatment time of 21 ± 18 s, where the large variation can be attributed to the high sensitivity to the environment (*i.e*., temperature, humidity, pressure) while the halogen vapor treatment is carried out. In fact, the environmental conditions may influence the grain boundaries of the crystallites, affecting thus the percolation threshold and therefore such conditions must be precisely controlled. Employing two different electrode geometries (*i.e*., in-line and in-square; see inset of Figure 2e and Supporting Information section S5), the electrical anisotropy was assessed by sequentially rotating the electrodes at 10 random angles between 0 < φ < 2π. A sheet conductance of σ_sheet,line_ = 0.27 ± 0.03 mS and σ_sheet,square_ = 0.26 ± 0.01 mS, for the in-line and in-square geometry, was calculated. Interestingly, BL films of α-ET_2_I_3_ manifest exceptional long-term stabilities, since a typical sample prepared for a previous publication^6^ showed the same sheet conductance value of σ_sheet_ = 0.28 ± 0.02 mS after being stored under ambient conditions for 10 years. These values are also in excellent agreement with the ones measured shortly after sample preparation.

### 2.3 Electrothermal Response and Temperature Coefficient of Resistance

**Figure 3:**
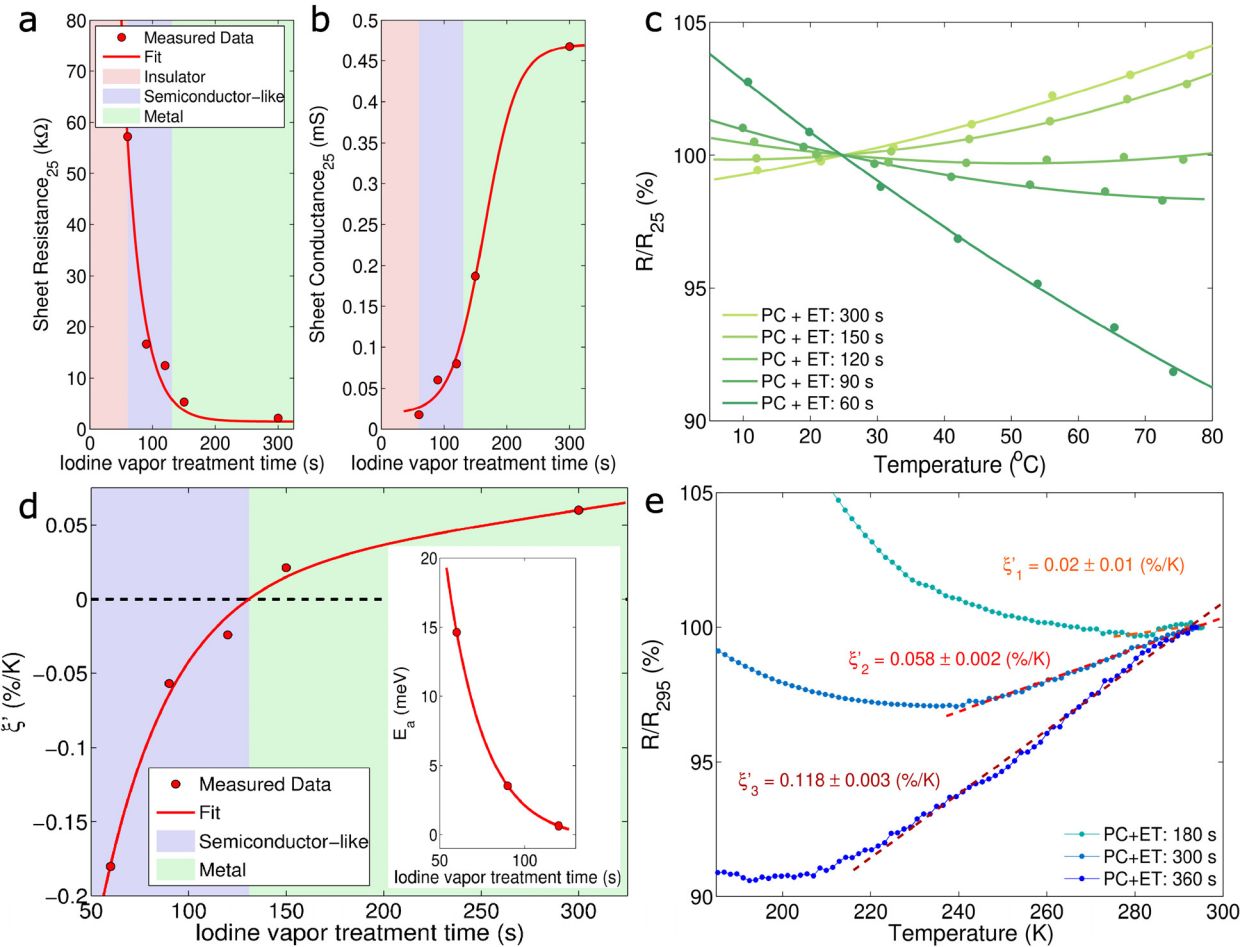
Electrothermal characteristics of BL films. (a) Sheet resistance and (b) sheet conductance of films as a function of iodine vapor treatment time. Continuous lines correspond to fits employing the exponential decay and Boltzmann sigmoid dependence. (c) Temperature dependence and best fits to second-order polynomial for resistance of α-ET_2_I_3_ BL films measured under ambient conditions at different iodine vapor treatment times. (d) Corresponding first-order temperature resistance coefficient ξ′ as a function of iodine treatment time. The inset shows the activation energy (*E*_a_) extracted for the semiconductor-like curves. (e) Relative resistance change of BL films measured in a vacuum and low temperature with corresponding first-order temperature resistance coefficients.

From the conducting point of view, the two main ingredients of α-ET_2_I_3_ BL-films are (i) polycarbonate, which is electrically insulating with a relatively large optical bandgap (*E*_g_ = 4.12 ± 0.01 eV)^30^ and a very low electrical conductivity (σ_PC_ = 10^–12^–10^–10^ S/cm)^31^ due to a broken π-conjugation between the phenyl rings in its chemical structure, and (ii) α-ET_2_I_3_ crystallites, which behave as an organic 2D metal at room temperature.^22^Figure 3a shows the electrical sheet resistance of BL films measured at different iodine vapor treatment times. Here, the resistance follows a clear exponential decay with a typical constant of τ_exp_ = 28 ± 14 s. Its sheet conductance, on the other side, can be approximated using a Boltzmann sigmoid; it starts at very small values, corresponding to a region limited by the low electrical conductivity of the polycarbonate, and crosses a state of intermediate conductance with a typical time constant of τ_sigmoid_ = 26 ± 10 s and finally stabilizing at a high conductance state characterized by σ_high_ = 0.47 ± 0.29 mS, for treatment times longer than 300 s. This value is in good agreement with the σ_0_ = 0.66 ± 0.15 mS, extracted above, by employing the percolation theory.

To gather information about the nature of charge transport mechanisms (*i.e*., semiconductor-like or metallic), the temperature response of resistance is typically used. Different samples of BL-films with increasing iodine vapor treatment times were mounted in a home-built measurement chamber that allows temperature sweeps, under an ambient atmosphere, in a range of 10 °C < *T* < 80 °C, while measuring the electrical resistance evolution in a Kelvin probe or 4-wire configuration. All measured V/I curves exhibit extraordinary linearity with values extracted in the linear fits equal or better than *R*^2^ = 0.9998. This reflects the very high homogeneity of the BL films as it was expected, since the effects of contact resistances are efficiently removed by the four probe technique, and the Ohms-law is valid for metals, semiconductors, and even insulators (*i.e*., very high resistivity as for instance PC; see also the Supporting Information for a complete set of temperature-dependent V/I characteristics, section S6). The change of relative resistance (*R*/*R*_25_) was fitted with the function *R* = *R*_25_ [1 + ξ′ (*T* – *T*_0_) + ξ″(*T* – *T*_0_)^2^] that permits to extract first (ξ′) and second (ξ″) order temperature resistance coefficients, having *T*_0_ = 25 °C. At low iodine treatment times (<90 s), a clear semiconductor-like temperature response was extracted. With increasing iodine vapor treatment times, the linear temperature resistance coefficient ξ′ approaches zero and becomes positive. Figure 3d allows extracting the semiconductor-like metal transition time at *t*_SM_ = 131 ± 10 s from the iodine vapor treatments. This result agrees with the activation energy *E*_a_ obtained by employing an Arrhenius type of temperature dependence for samples treated with halogen vapors, with an exponential decrease approaching zero at *t*_SM_ as the percolation pathways grow larger (inset of Figure 3d and Supporting Information section S6). The values of this activation energy are smaller than *k*_B_*T* at room temperature (*i.e*., *k*_B_*T* ≈ 26 meV) and much smaller than that from the monomer bandgap of neutral ET (*vide infra*), explaining the dc-conductivity of the composite at room temperature. This activation energy reflects the difference between the acceptor level (iodine) and the highest occupied molecular orbital (HOMO) of ET.^32^

A similar experiment, carried out in a cryostat under vacuum, allows reaching much lower temperatures down to 190 K. Figure 3e shows the temperature response of the relative resistance (*R*/*R*_295_) measured for three samples with iodine treatment times of *t* = 180, 300, and 360 s. In agreement with the results discussed above, all three curves exhibit a clear positive first-order temperature resistance coefficient of ξ′_180s_ = 0.02 ± 0.01 (%/K), ξ′_300s_ = 0.058 ± 0.002 (%/K), and ξ′_360s_ = 0.118 ± 0.003 (%/K) close to room temperature. The most conductive film, treated at *t* = 360 s, was measured down to *T* = 10 K exhibiting a relative conductivity approaching zero (see Supporting Information section S6). For single crystals of α-ET_2_I_3_, a sharp metal–insulator (MI) transition was previously reported at *T*_MI_ = 135 ± 1 K.^22^ The thermal expansion coefficient, [λ = Δ*L*/(*L*_0_Δ*T*)], found in single crystals, exhibited a comparable value in both the **a** and **b** directions, with λ_a_ = 60 ± 1 (10^–6^/K) and λ_b_ = 59 ± 1 (10^–6^/K), respectively,^33^ being very close to the value typically found for polycarbonate λ = 65–70 (10^–6^/K).^34^ Perpendicular to the conductive plane, (c* = **a × b**), the thermal expansion coefficient is significantly smaller with λ_c*_ = 25 ± 2 (10^–6^/K).^33^

Close to the MI-transition temperature, the anisotropic thermal expansion of α-ET_2_I_3_ crystals was previously studied by capacitive dilatometry cells as well as by X-ray diffraction.^33^ Data confirmed a first-order phase transition with a contraction along the donor stacks, the **a** direction, and an expansion along the **b**-direction, with Δ**a**/**a** = (−0.86 ± 0.15) × 10^–3^ and Δ**b**/**b** = (+1.90 ± 0.25) × 10^–3^, respectively. The smallest contraction was found perpendicular to the conductive plain with Δc*/c* = (−0.20 ± 0.03) × 10^–3^. It was previously suggested that this may result in internal stress related to Coulomb forces associated with a redistribution of charge in the unit cell.^33^ Simple percolation theory, assuming an insulating matrix with conductive fillers fails to explain such a dramatic difference. Upon cooling the polymeric matrix contracts, the distance between conductive elements would decrease, and hence the resistance of the composite should continue to decrease.^24^ The observed MI-transition is much broader compared to the one reported for single crystals, so we can conclude that these large differences can be attributed to the mismatch and the high anisotropy of thermal expansion coefficients between the molecular metal and the polymeric matrix. At very low temperatures, a similar phenomenon was attributed to a significant shift of the superconducting transition in β-ET_2_I_3_ microcrystals in the polymer.^35,36^ Variations in the vertical *d*-spacing (Δc*/c*) observed in similar hydroresistive BL films were exploited to prepare highly sensitive and fully reversible humidity sensors.^8^ On the other hand, close to room temperature, the thermal expansion of crystals of the molecular metal α-ET_2_I_3_ and the polymeric PC matrix show similar values and are therefore the ideal candidate to fabricate flexible and soft sensors applicable under human physiological conditions.^19,37^

Summarizing, electrothermal characterizations of BL films at different iodine vapor treatment times allow extracting temperature resistance coefficients of such films exhibiting a clear insulator, or semiconductor-like, to metal transition. This is particularly interesting since both starting components on their own exhibit either insulating or metallic electrical properties close to room temperature and the semiconductor-like behavior emerges as the composite forms. This transition can be attributed to the importance of grain size and boundaries in composites, which can influence the overall electrical properties considerably and allow tuning the temperature resistance coefficient from semiconductor-like (*i.e*., ξ′ < 0) to semimetal (*i.e*., ξ′ = 0) and all the way to metal-like (*i.e*., ξ′ > 0).

### 2.4 Optical Response and Extraction of Bandgap of BL

**Figure 4:**
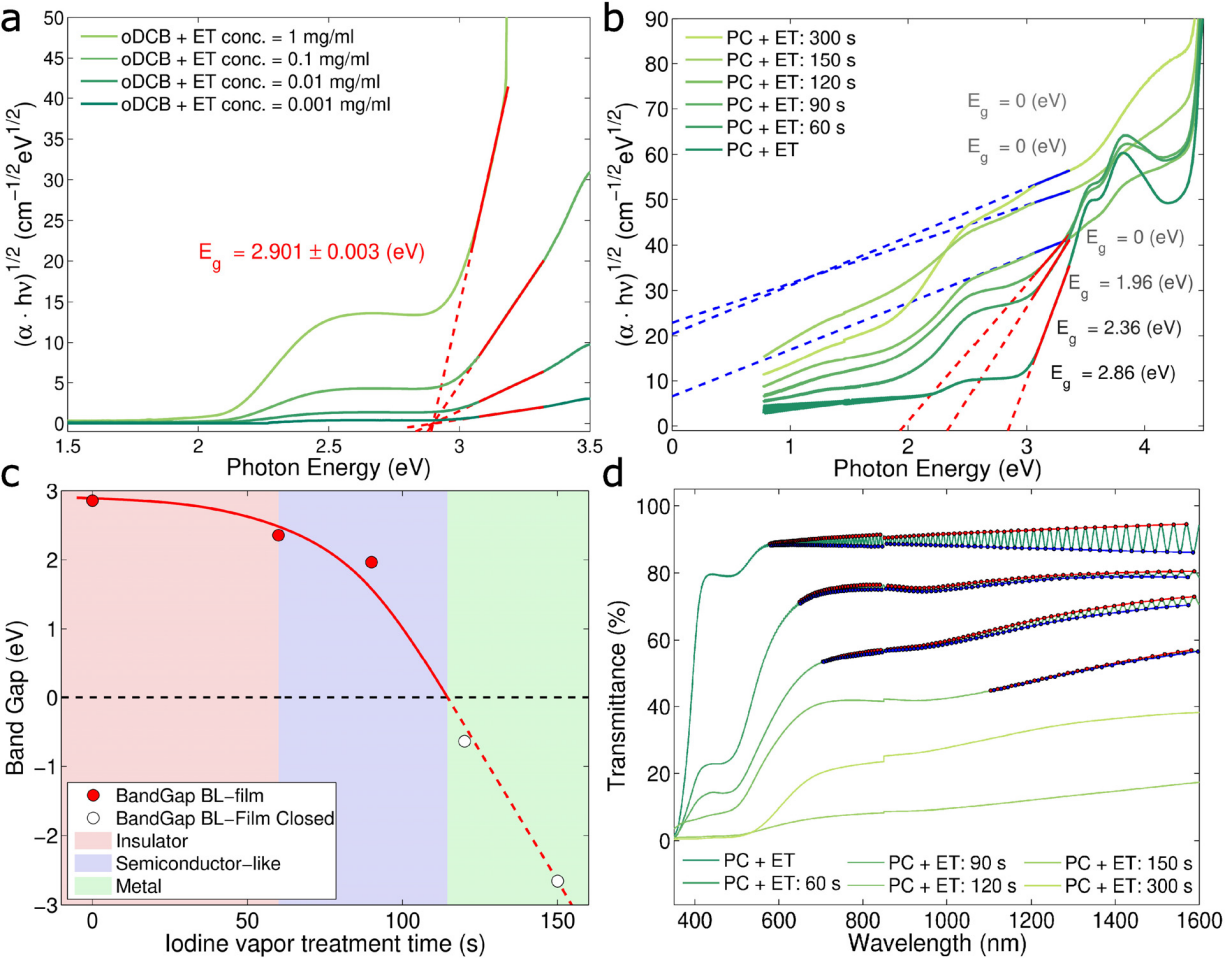
Optoelectronic characteristics of ET in solution and of solid-state BL films. (a) Tauc plots calculated by employing data obtained in transmission spectroscopy with ET dissolved in 1,2- dichlorobenzene (oDCB) at different concentrations and extraction of the monomer band gap. (b) Tauc plots of ET in solid-state films at different iodine vapor treatment times and extraction of the optical bandgap for the BL nanocomposite. (c) Corresponding bandgap of BL nanocomposite as a function of iodine treatment time with a clear insulator, semiconductor-like, and metal transitions. Transmission spectra for films at different iodine vapor treatment times and corresponding interference patterns at high wavelengths.

Differences between insulators and metals can be also studied by examining the material’s optical properties, *i.e*., the absorbance spectrum. The absorbance spectrum of neutral ET dissolved in 1,2-dichlorobenzene (*o*DCB) was measured employing different concentrations (*c* = 0.001, 0.01, 0.1, and 1 mg/mL). The Tauc plot (see Supporting Information section S7), with an exponent chosen to be 1/2, typical for indirect bandgap semiconductors or amorphous substances, was used to extract the monomer bandgap of ET.^38,39^ The choice is reasonable since the ET molecules are dissolved in *o*DCB. The shoulder at about 2 eV corresponds to the ET_2_ dimers, often observed in strongly correlated π-conjugated systems such as ET.^38,40^ The monomer bandgap of ET was extracted to be about *E*_g_ = 2.901 ± 0.003 eV and is in good agreement with the literature values.^22,38^ A similar experiment was carried out studying films in the solid-state, where the neutral ET molecule can be considered as a solid-state solution in polycarbonate. Again, the ET_2_ dimer shoulder, corresponding to sub-bandgap states, was observed in the absorbance spectra. In samples at the pristine state, *i.e*., before halogen vapor treatment, the Tauc plot exhibited a similar value for the bandgap, *E*_g_ = 2.86 eV, which is similar to the monomer value extracted from a liquid solution. With increasing iodine vapor treatment time, the extracted optical bandgap decreases and disappears at treatment times greater than *t* = 114 ± 15 s (see Figure 4c), which is in good agreement with the temperature resistance coefficient shown in Figure 3d. Remarkably, with the different iodine treatment, it is possible to obtain semiconducting BL films with optical bandgaps ranging from 0 to 2.9 eV. In comparison to that, the shoulder at about 2 eV does not alter considerably with increasing iodine treatment times (see also Supporting Information section S7). This observation further supports the hypothesis that this shoulder can be attributed to sub bandgap states formed by the above-mentioned dimers. The ET molecule in its neutral form is not flat, but after a charge transfer, as a radical cation ET^•+^, the molecule gets planar which is energetically favored. The formation of dimers in solution, or in the solid-state as well as the crystallization into well-ordered periodical structures, can lead to energy level splitting typically observed as a red shift in the absorbance spectra.^40^ As anticipated in the Introduction, a major difference between a semiconductor and a metal is its ability to transmit light. Once the optical bandgap closes, the transmission spectra of the films decrease considerably. Optical interference is often observed in thin films when film thickness exceeds the wavelength of the incident light.^41^ This phenomenon was used to estimate the full film thickness, *i.e*., polycarbonate matrix, plus a topmost conducting network of a molecular conductor leading to a value of *d* = 29 ± 2 μm (see also Supporting Information section S8). These values are comparable to measurements carried out by using a micrometer gauge which exhibited values of *d* = 28 ± 1.6 μm. Interestingly, the interference pattern decreases as the bandgap closes and disappears once the BL film turns metallic, in agreement with iodine treatment times greater than 120 s. Therefore, the optical bandgap of BL films can be efficiently tuned by controlling the iodine vapor treatment time. This allows designing tailor-made materials with a tunable and similar bandgap, extracted both by electrothermal and optical characterizations.

To demonstrate potential applications of the developed materials, a set of prototypes, exploiting piezoresistivity^6^ and pyroresistivity,^7^ have been prepared. Prototype no. 1 comprises a BL film with α-ET_2_I_3_ as an electrically active component wrapped around a plastic tube able to detect pressure changes down to 1.33 ± 0.03 Ω/mbar. Prototypes no. 2 and no. 3 consist of semiconductor-like (ξ′ = −0.18%/K) and metallic (ξ′ = 0.12%/K) BL films mounted on the plastic tube. Both sensor elements, even though only surface mounted, exhibit superior performance compared to a commercial platinum sensor arranged in direct contact with the fluid inside the tube in an invasive fashion. Similar prototypes could be used to precisely control pressure and temperature in a variety of scenarios including, for instance, intraocular eye surgery or the removal of a cataract. See Supporting Information section S9 for more details on the prototype development.

## 3 Conclusion

In summary, the optoelectronic properties of insulating polycarbonate/molecular two-dimensional metal (α-ET2I3) composites have been studied in detail. A tunable electrical bandgap, monitored by temperature-dependent resistance measurements agrees well with the optical bandgap extracted by optical absorption spectroscopy making it possible to obtain semiconducting BL films with tunable bandgaps ranging from 0 to 2.9 eV. Similar exponential decay constants, obtained from electro-thermal, optical, and micro-composition measurements hint towards a diffusion-based formation of the topmost conducting layer and a phase separation of the (nano)microcrystals of the conducting α-ET2I3 salts, which is responsible for the emergent insulator semiconductor-like metal transition. These findings are not limited to the particular material used in this study and will allow designing novel materials with easy process ability and tailor-made mechanical, electrical, optical, and thermal properties, particularly interesting for human health care and remote medicine applications as showcased with a few prototypes. Research in this direction is in progress using different molecule-based materials.

## 4 Materials and Methods

### 4.1 Materials and Sample Preparation

Bis(ethylenedithio)-tetrathiafulvalene (BEDT-TTF or ET), poly(bisphenol A-carbonate) (PC) in pellets (average MW ∼64000), 1,2-dichlorobenzene (oDCM), and dichloromethane (DCM) were purchased from Sigma-Aldrich and used without further purification. The preparation of BL films comprises a two step procedure: (i) Step one consists of casting a hot solution of PC (98 wt %) and BEDT-TTF (2 wt %) in oDCB (concn = 20 g/L) into a Petri dish at *T* = 130 °C, and evaporation of the solvent, that leads to a film with molecularly dispersed BEDT-TTF and a total thickness of about 20–30 μm. The sample was kept in the oven for 20 min to let the solvent evaporate. (ii) Step two consist of film treatment with halogen vapors and the formation of the polycrystalline conducting layer. A glass flask containing the binary system of the I_2_/CH_2_Cl_2_, saturated solution, was enclosed inside a chamber with a thermostat and left to equilibrate at 25 °C for 45 min. After that, the samples were treated with increasing times exposing them to vapors of solvent and oxidant by placing them as a lid on the top of the flask.

### 4.2 Electrothermal, Optical, and Chemical Characterization

If not otherwise stated, all electrical measurements were performed using four-wire dc-resistance measurements employing a constant current up to about *I* = 1 μA to prevent Joule heating (with a Keithley model 2612 or 2450). For low-temperature measurements, samples were electrically contacted with conductive graphite paste (Dotite XC-12) and mounted to a Cryostat from LakeShore (model CRX-6.5K) operated with a Lakeshore temperature controller (model 336) at a vacuum of *P* ≈ 10^–7^ mbar. Slow temperature sweeps in the range of 3 K/min were carried out to ensure thermal stabilization of the mounted samples. All remote measurements were performed using homemade Python measurement routines, and data analysis was done with MATLAB. UV–vis spectroscopy was carried out employing a Jasco V-780 spectrometer. SEM-EDX microanalysis was done at low vacuum (*P* = 60 Pa) using a Quanta FEI 200 FEG-ESEM microscope. Images were acquired at 10 kV. The air pressure applied in testing prototype no. 1 was controlled with a WIKA low-pressure controller CPC 2000.

## Supporting Information Available

Additional information given in sections S1, energy-dispersive X-ray spectroscopy (EDX) analysis; S2, energy-dispersive X-ray spectroscopy (EDX) analysis, cross section; S3, estimated evolution of α-ET_2_I_3_/(α-ET_2_I_3_+PC), ratio of the topmost polycrystalline network; (S4) scanning electron microscope (SEM) images; S5, electrical anisotropy of topmost conducting layer; S6., electro-thermal response, temperature dependence of voltage–current characteristics; S7, optical response and extraction of bandgap and sub-bandgap states; S8, estimation of total film thickness by interference and a micrometer gauge; and S9, prototype development, pressure sensor and temperature sensor (PDF)

## Abbreviations

BEDT-TTF or ET: bis(ethylenedithio)tetrathiafulvalene
PC: poly(bisphenol A-carbonate)
BL: bilayer
SEM: scanning electron microscopy
EDX: energy-dispersive X-ray
*o*DCB: *ortho*-dichlorobenzene
DCM: dichloromethane
HOMO: highest occupied molecular orbital
MI: metal–insulator
