# Supplementary material for: Emergent Insulator–Metal Transition with Tunable Optical and Electrical Gap in Thin Films of a Molecular Conducting Composite
Source: ACS Appl Electron Mater. 2022 May 11;4(5):2432–41. doi: 10.1021/acsaelm.2c00224 (PMC9134344; doi:10.1021/acsaelm.2c00224)
Supplement: Supplementary file 1 — el2c00224_si_001.pdf [file el2c00224_si_001.pdf]

Supporting Information

Title:

Emergent Insulator-Metal Transition with Tunable Optical and Electrical Gap in Thin Films of a Molecular Conducting Composite

*Author(s), and Corresponding Author(s)**

Raphael Pfattner,[1,2,*] Elena Laukhina,[2,*] Jinghai Li,[1] Rossella L. Zaffino,[1] Núria Aliaga-Alcalde,[1,3] Marta Mas-Torrent,[1,2] Vladimir Laukhin,[1,2,3] and Jaume Veciana[1,2*]

Corresponding author email:

rpfattner@icmab.es, laukhina@icmab.es and vecianaj@icmab.es

Table of contents:

S1.) Energy-Dispersive X-ray spectroscopy (EDX) analysis S2

S2.) Energy-Dispersive X-ray spectroscopy (EDX) analysis – Cross section S10

S3.) Estimated evolution of α-ET_2I_3/(α-ET_2I_3 + PC) – ratio of the polycrystalline network S11

S4.) Scanning electron microscope (SEM) images S13

S5.) Electrical anisotropy of topmost conducting layer S15

S6.) Electro-thermal response - temperature dependence of voltage-current characteristics S17

S7.) Optical response and extraction of bandgap and sub-bandgap states S22

S8.) Estimation of total film thickness by interference and micrometer gauge S23

S9.) Prototype development: Pressure sensor and Temperature sensor S25

S1.) Energy-Dispersive X-ray spectroscopy (EDX) analysis

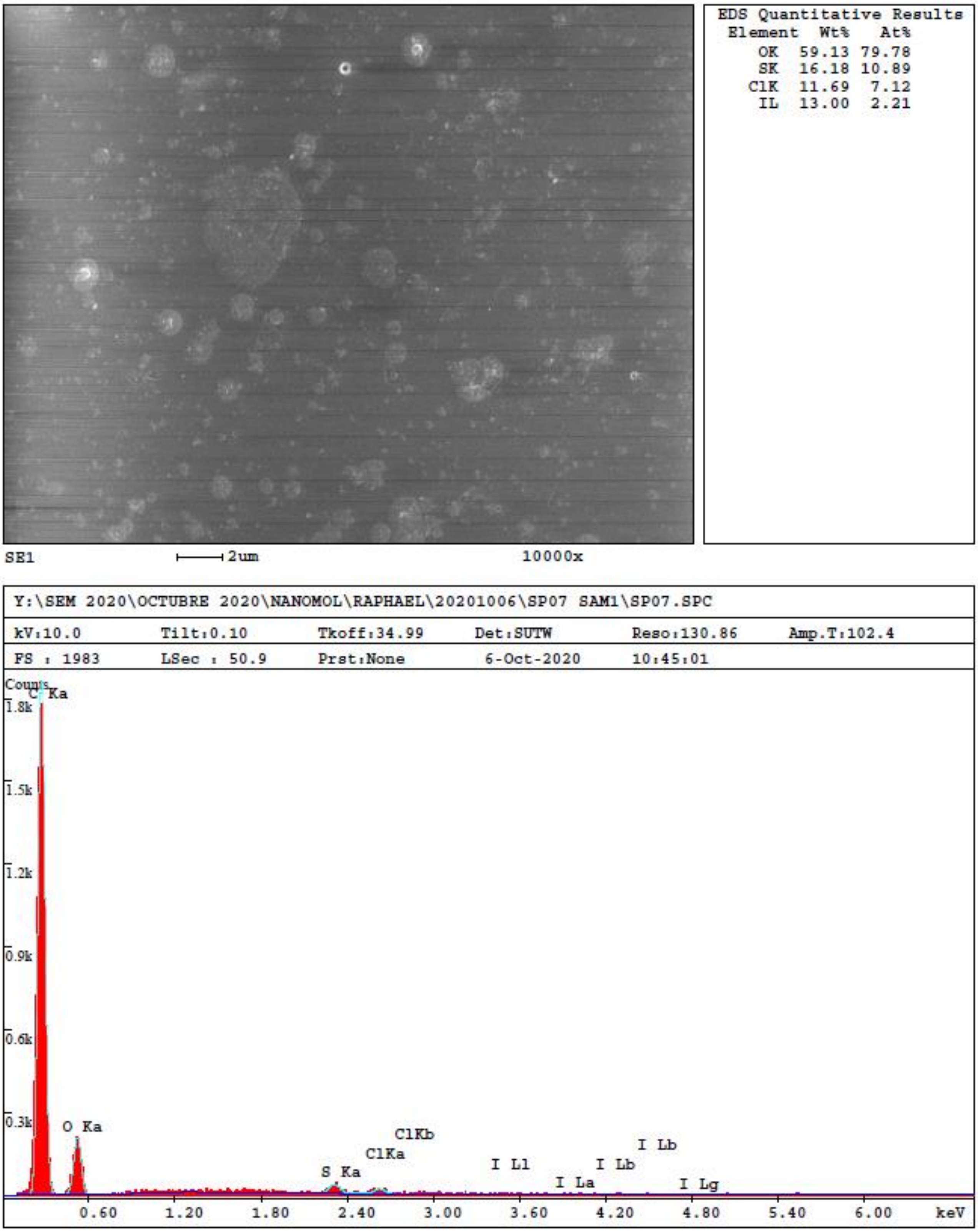

Figure S1: Energy-dispersive X-ray spectroscopy (EDX). Carried out on pristine PC + ET, front side of sample.

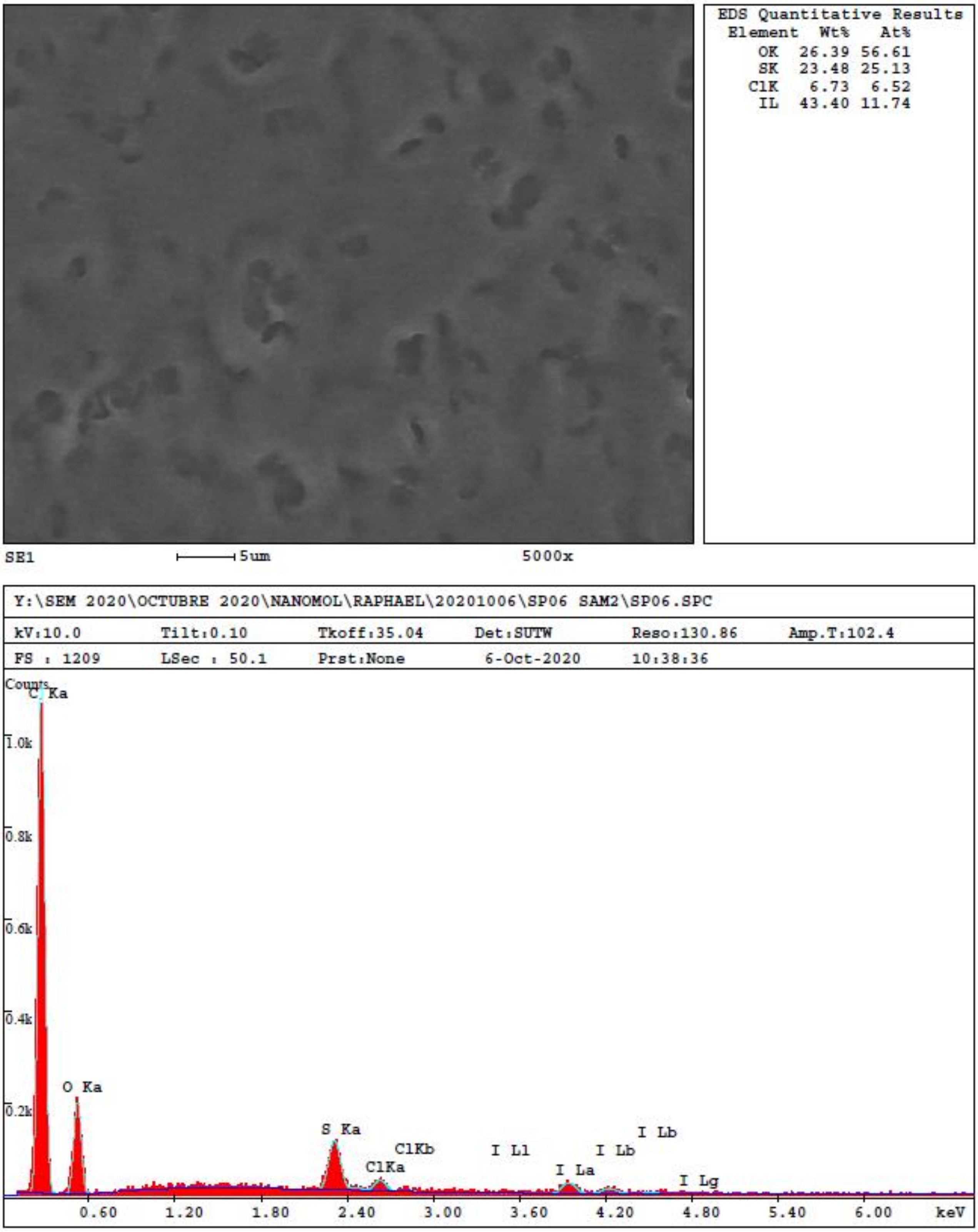

Figure S2: Energy-dispersive X-ray spectroscopy (EDX). Carried out on PC + ET and 30 s halogen vapor treatment, front side of sample.

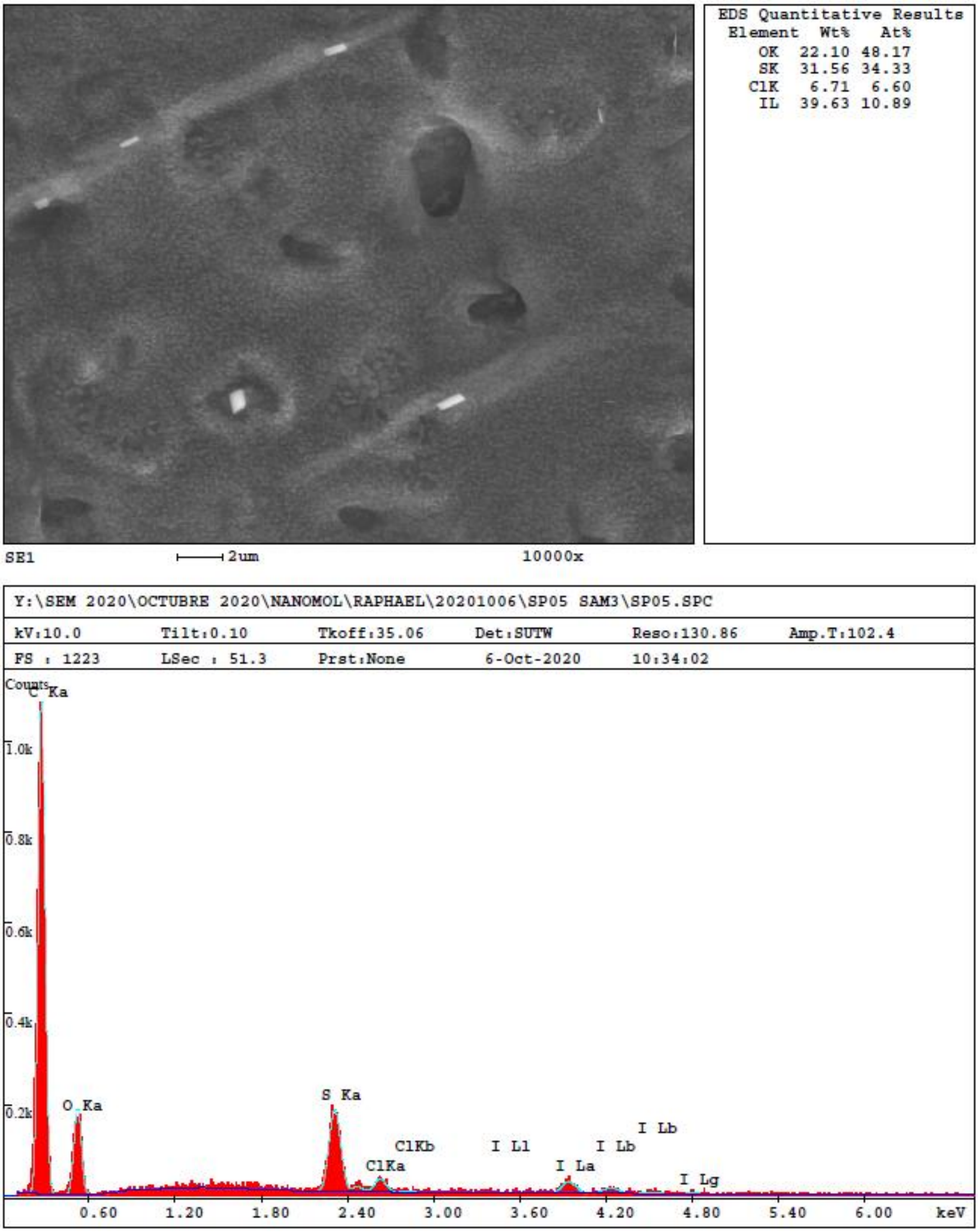

Figure S3: Energy-dispersive X-ray spectroscopy (EDX). Carried out on PC + ET and 60 s halogen vapor treatment, front side of sample.

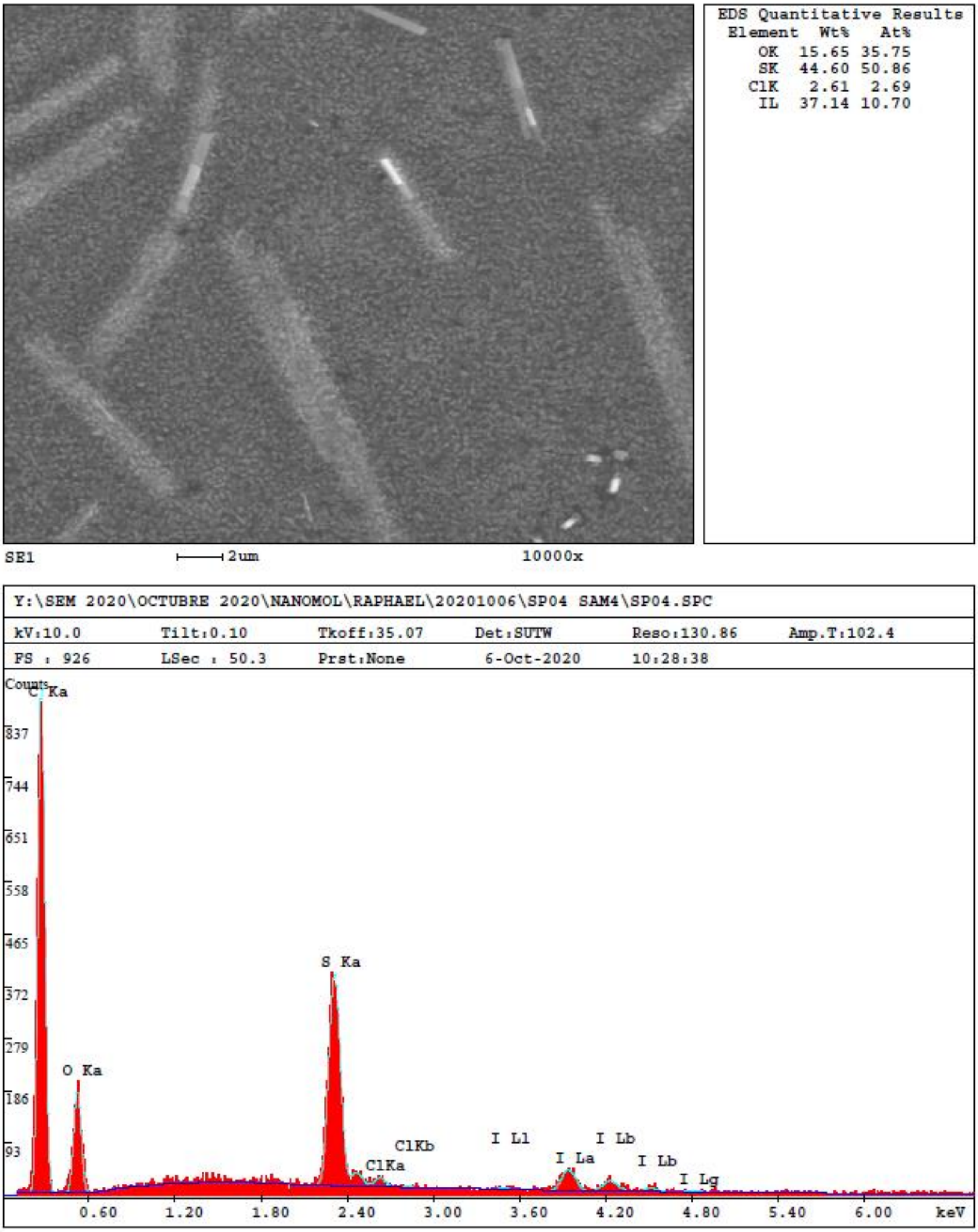

Figure S4: Energy-dispersive X-ray spectroscopy (EDX). Carried out on PC + ET and 90 s halogen vapor treatment, front side of sample.

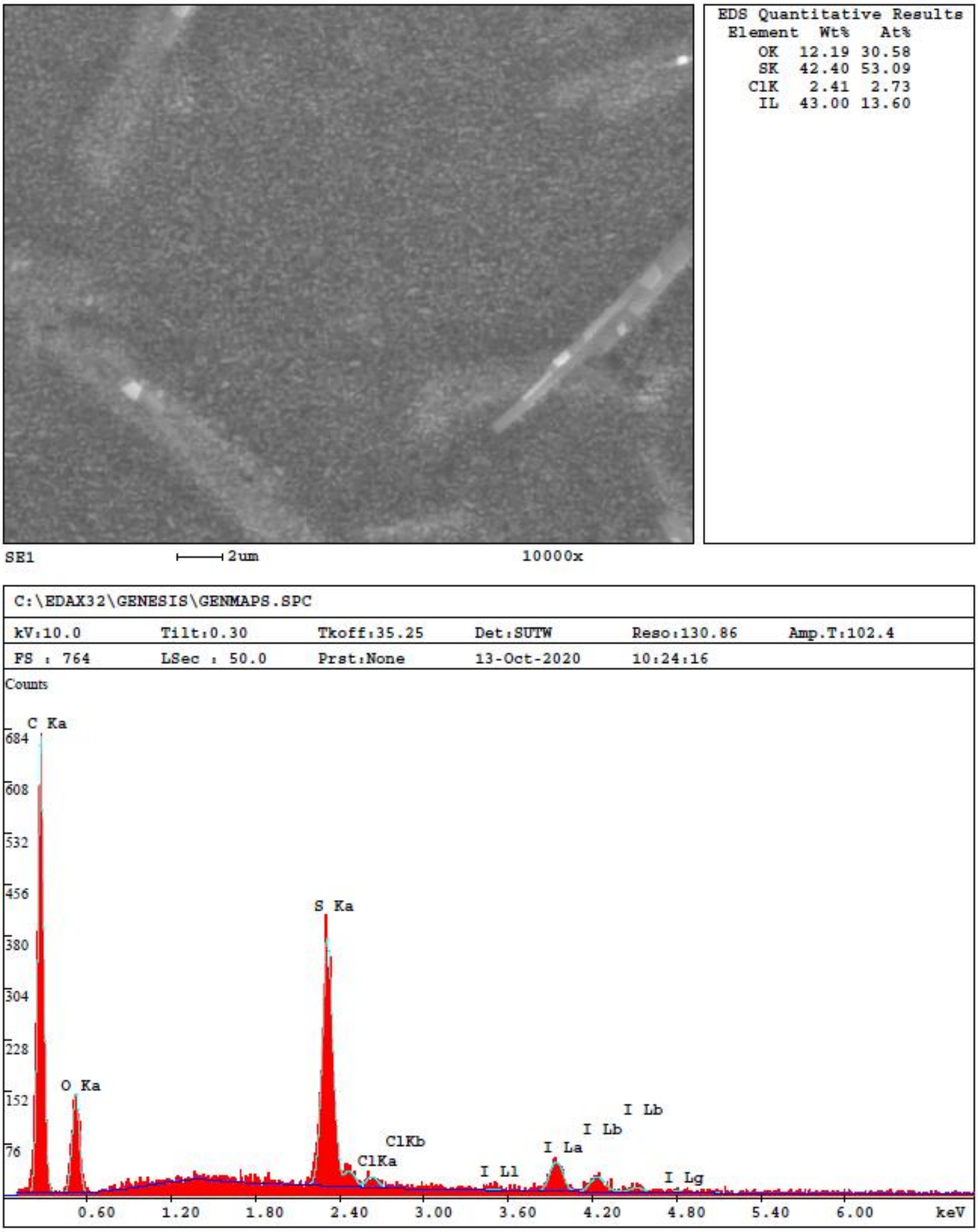

Figure S5: Energy-dispersive X-ray spectroscopy (EDX). Carried out on PC + ET and 120 s halogen vapor treatment, front side of sample.

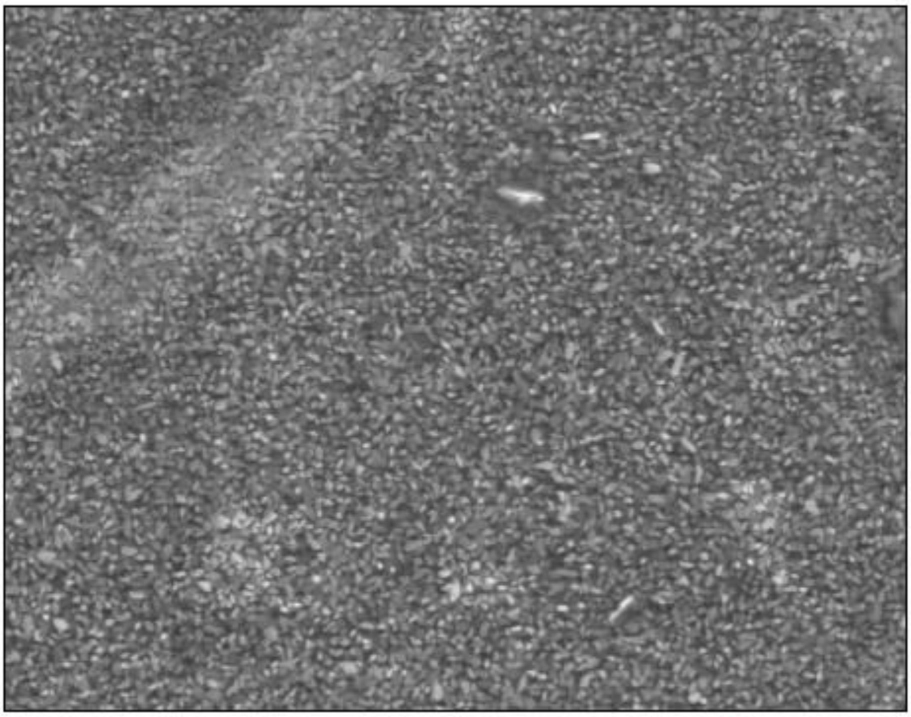

EDS Quantitative Results		
Element	Wt%	At%
OK	9.70	26.22
SK	40.73	54.93
ClK	2.23	2.72
IL	47.33	16.13

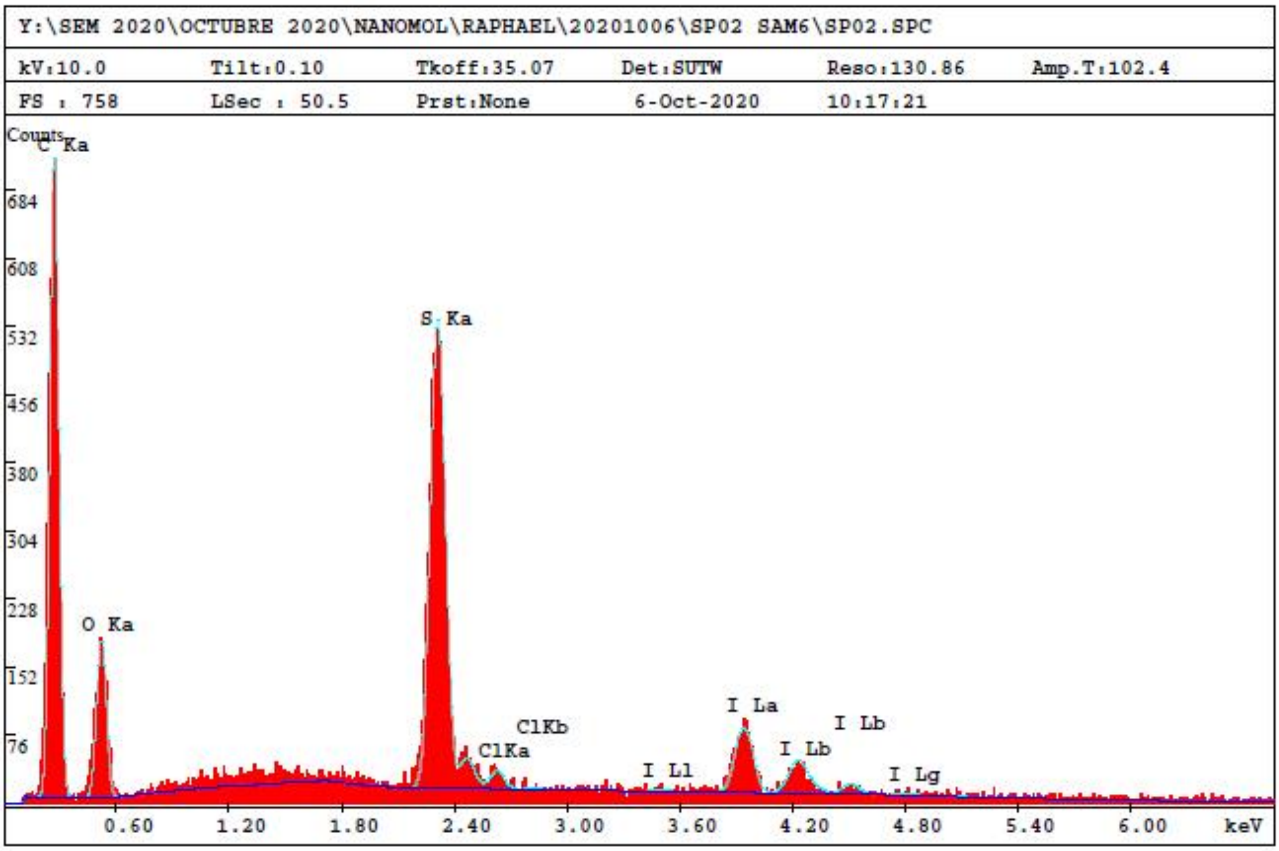

Figure S6: Energy-dispersive X-ray spectroscopy (EDX). Carried out on PC + ET and 150 s halogen vapor treatment, front side of sample.

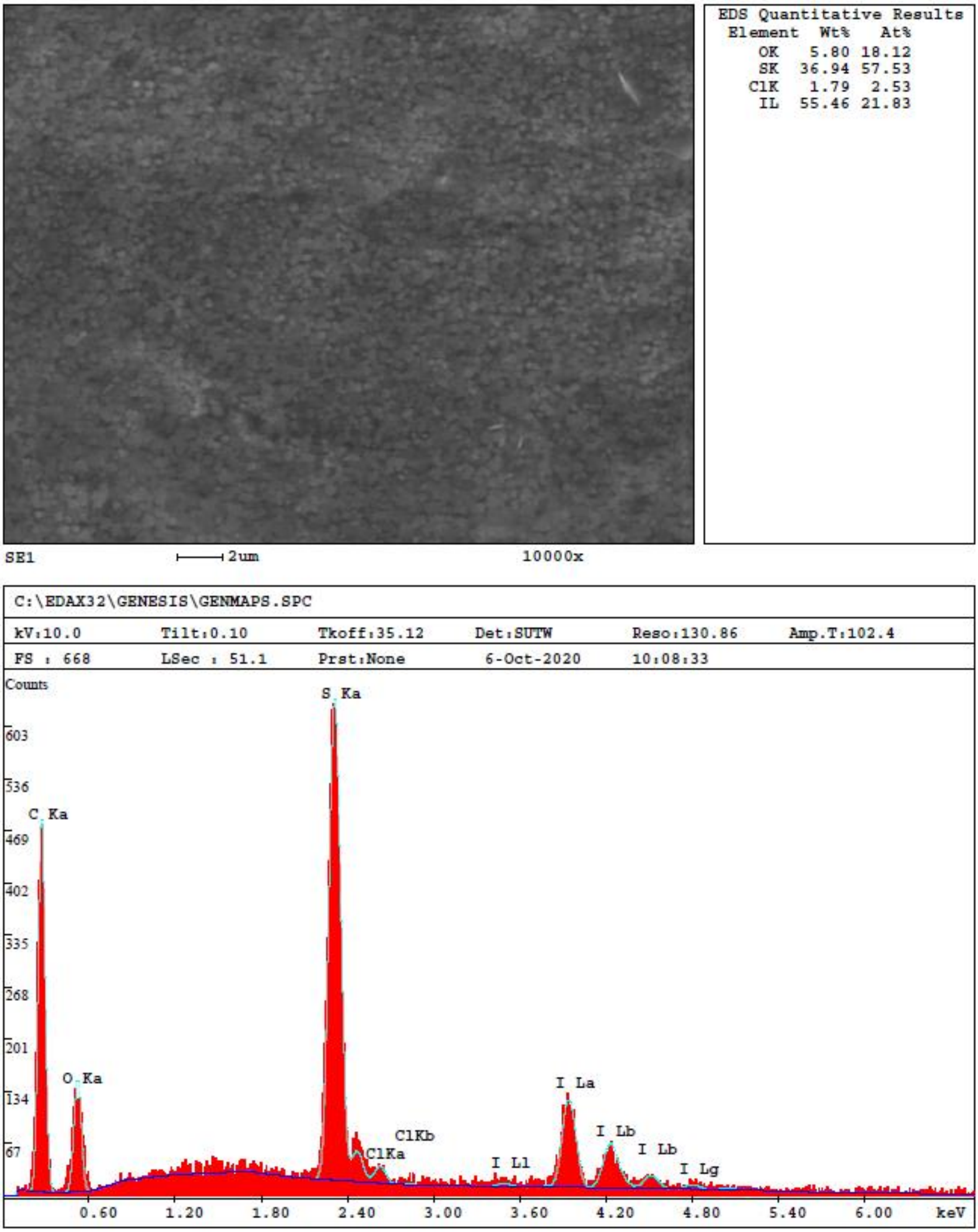

Figure S7: Energy-dispersive X-ray spectroscopy (EDX). Carried out on PC + ET and 300 s halogen vapor treatment, front side of sample.

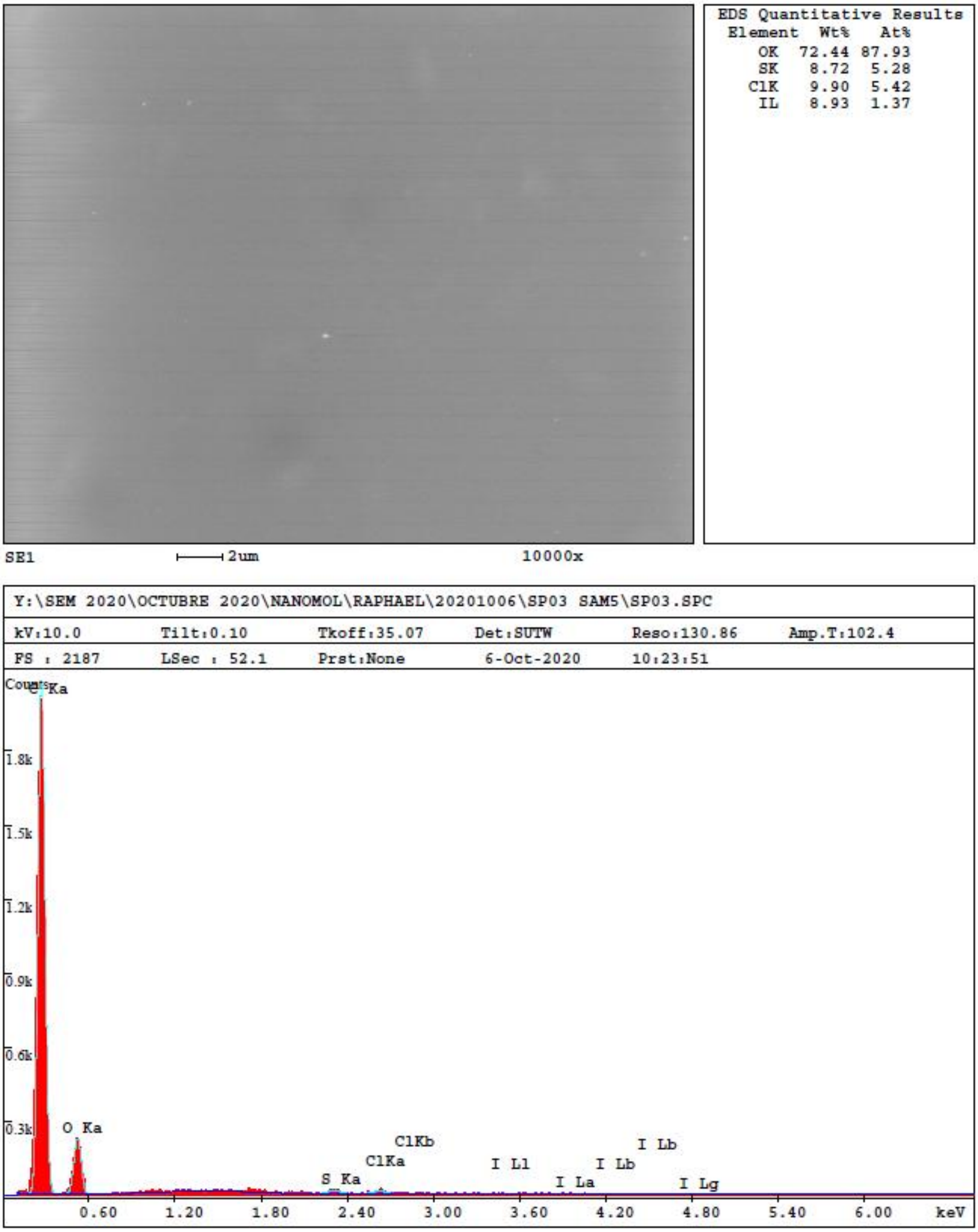

Figure S8: Energy-dispersive X-ray spectroscopy (EDX). Carried out on PC + ET and 120 s halogen vapor treatment, back side of sample.

S2.) Energy-Dispersive X-ray spectroscopy (EDX) analysis – Cross section

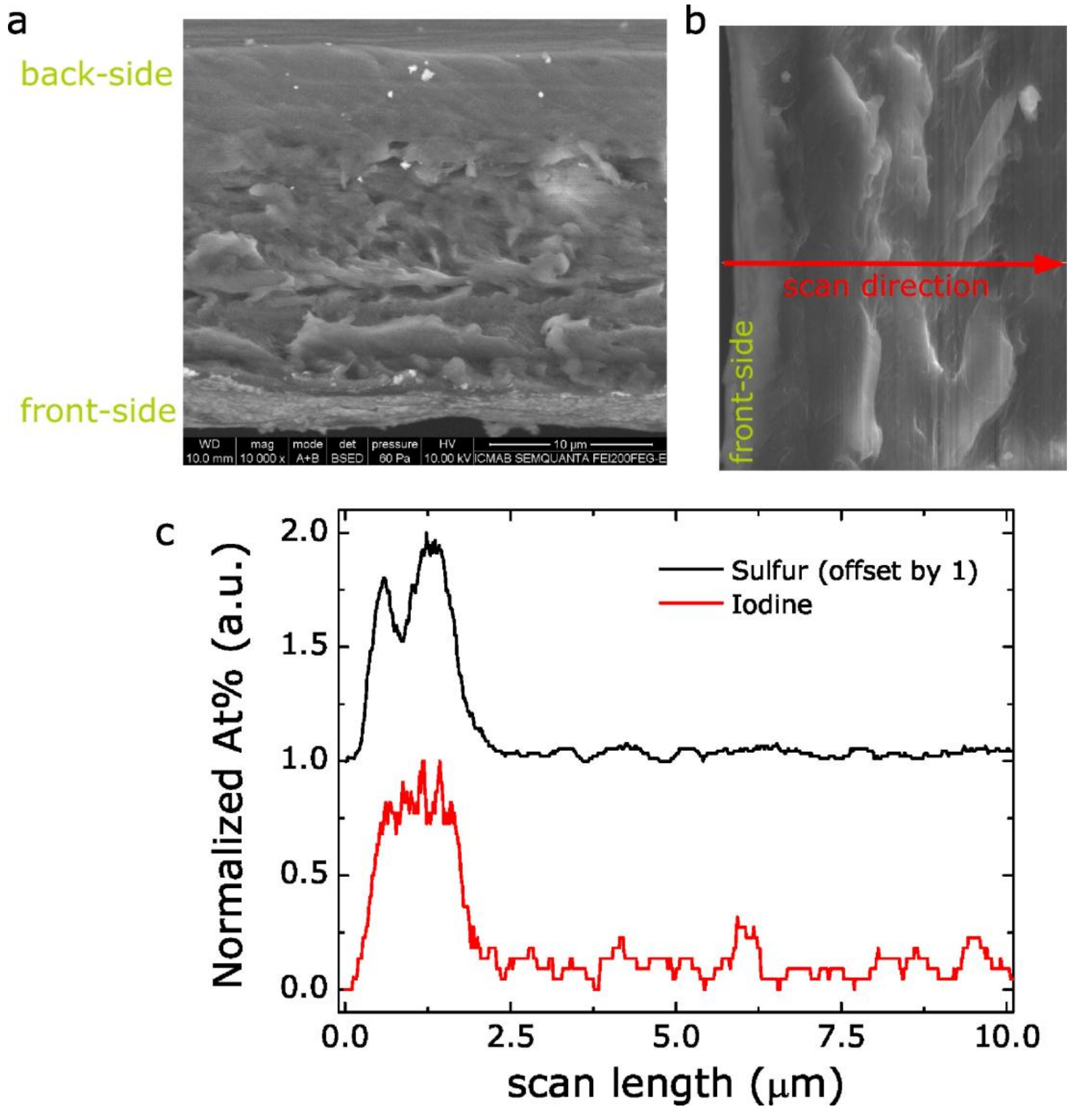

Figure S9: Energy-dispersive X-ray spectroscopy (EDX) on cross section of BL-film treated with iodine vapors for t = 300s. (a) Full cross section of sample, (b) first 10 µm close to front-side and (c) Normalized Atomic concentration recorded in a scan along the red arrow shown in (b).

S3.) Estimated evolution of α-ET_2I_3/(α-ET_2I_3 + PC) – ratio of the polycrystalline network

After solution casting, the pristine sample in the BL-film fabrication process consists of a solid-state solution of neutral ET donor molecules in a PC matrix. During the halogen treatment DCM is used to swell the polymeric matrix and this induces a slow diffusion process of neutral ET donor molecules and iodine. Thus, the formation of the topmost polycrystalline network in α-ET_2I_3 BL films is a diffusion limited crystallization process (*i.e.* the crystallization is faster than the diffusion). This is reasonable, and in fact a previous publication employing a pair of proteins with identical shells but different molecular masses exhibited an almost identical kinetic coefficient for crystallization, thus, indicating, diffusion-limited kinetics of crystallization. Data further suggests that this phase transition is valid for a broad class of materials ranging from small molecules to proteins.[1]

The atomic weight percent of iodine extracted employing EDX analysis as function of iodine vapor treatment time shown in **Figure 1a**, allows estimating the α-ET_2I_3/(α-ET_2I_3 + PC) – ratio by using two boundary conditions:

i) we know that at treatment times $t = 0$ s there is no iodine present in the BL-films, and it is fair to assume that the α-ET_2I_3/(α-ET_2I_3 + PC) – ratio equals zero;

ii) we also know that at treatment times $t > 300$s, the conductivity of BL films stabilizes as α-ET_2I_3 crystallites are fully connected in the polycrystalline network,[2] thus the α-ET_2I_3/(α-ET_2I_3 + PC) – ratio equals one.

The iodine EDX signal originates mostly from α-ET_2I_3 as the estimated stoichiometry is very similar for all treatment times as shown in **Figure 1b**. Figure S10a shows the estimated evolution of α-ET_2I_3/(α-ET_2I_3 + PC) - ratio at different iodine treatment times, which can be approximated to a first order within an error of 8 %, estimated employing residuals of the linear regression shown in Figure S10b.

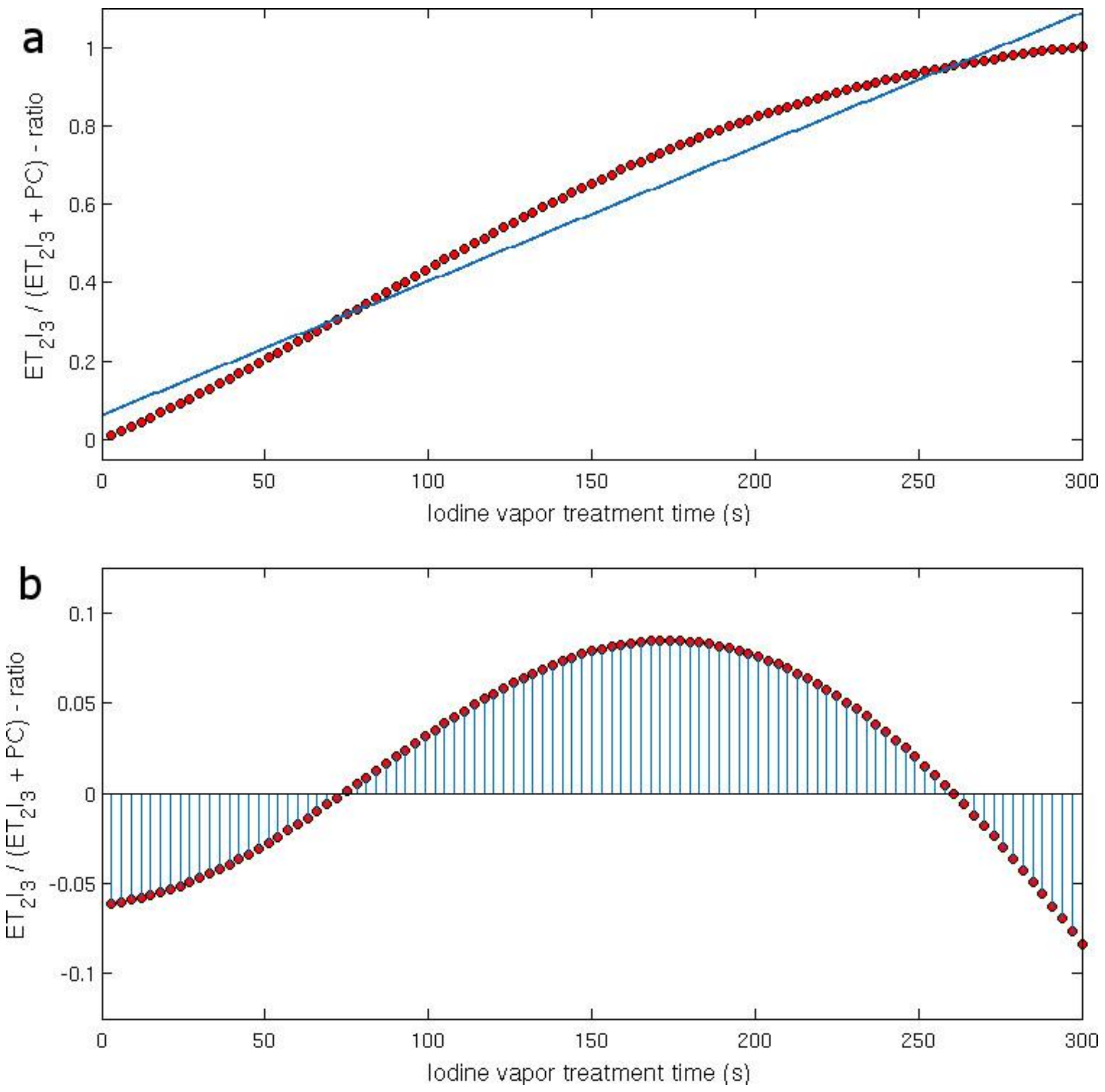

Figure S10: Estimation of α-ET_2I_3/(α-ET_2I_3 + PC) - ratio in topmost polycrystalline network of BL films. (a) α-ET_2I_3/(α-ET_2I_3 + PC) – ratio at different iodine treatment times fitted with a first order regression and (b) corresponding residuals.

S4.) Scanning electron microscope (SEM) images

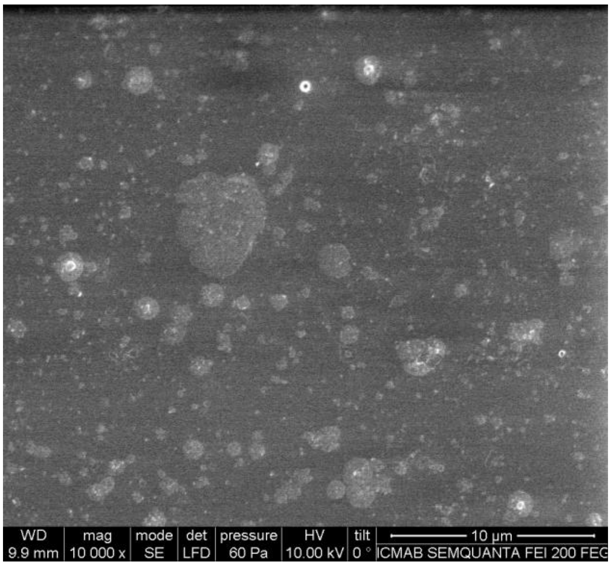

(a)

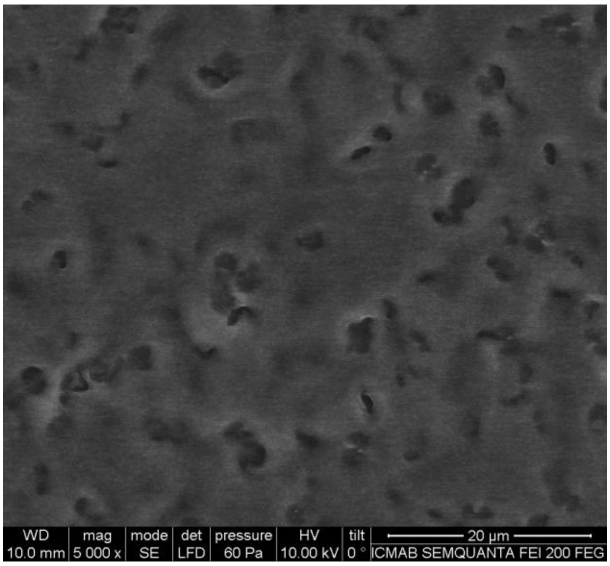

(b)

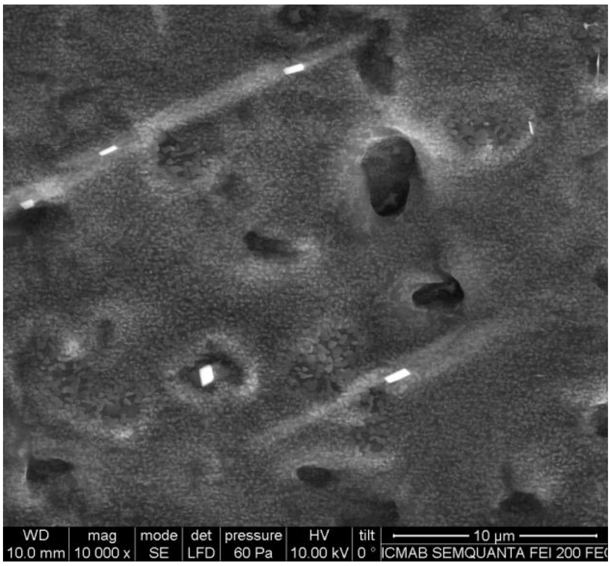

(c)

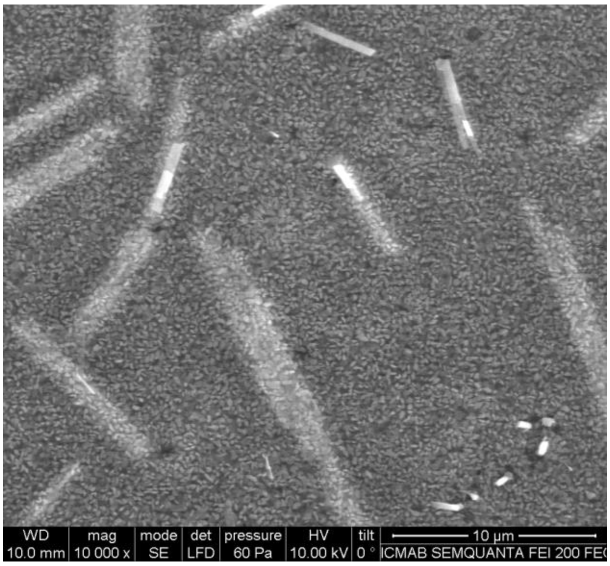

(d)

Figure S11: Scanning electron microscope (SEM) images taken in Low Vacuum Secondary Electron (LFD) mode. (a) Pristine PC + ET front side, (b) 30 s halogen vapor treatment front side, (c) 60 s halogen vapor treatment front side and (d) 90 s halogen vapor treatment front side.

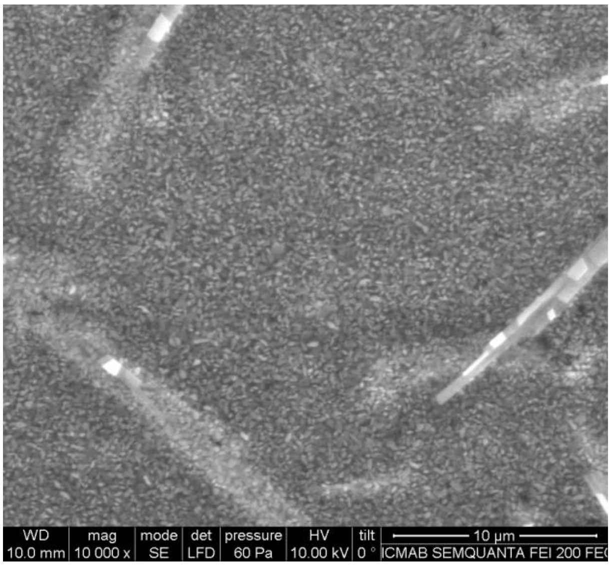

(a)

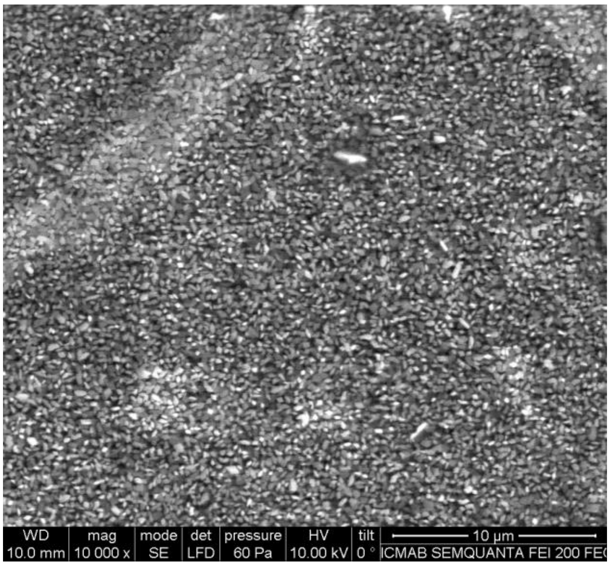

(b)

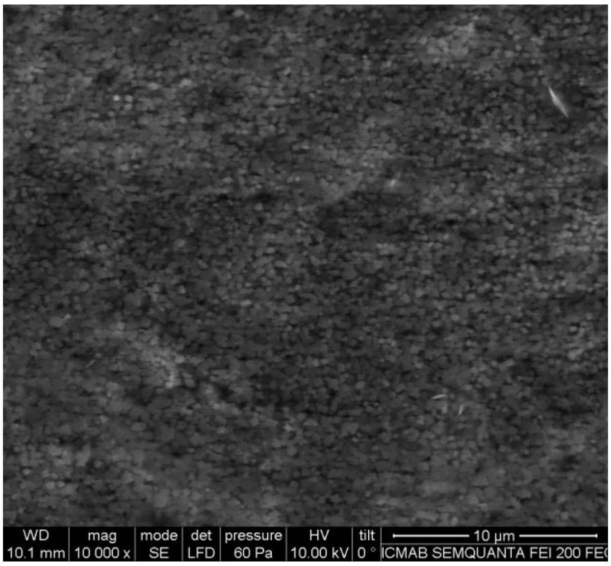

(c)

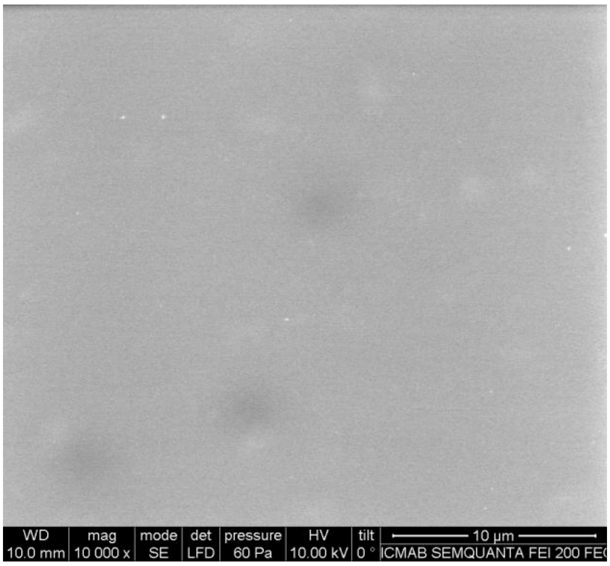

(d)

Figure S12: Scanning electron microscope (SEM) images taken in Low Vacuum Secondary Electron (LFD) mode. (a) 120 s halogen vapor treatment front side, (b) 150 s halogen vapor treatment front side and (c) 300 s halogen vapor treatment front side and (d) 120 s halogen vapor treatment back side.

S5.) Electrical anisotropy of topmost conducting layer

Figure S13 shows the two geometries used to extract the sheet resistance of BL films applying up to 1 μA of current between contacts I_1^- and I_4^+, while measuring the voltage drop between contacts V_1^- and V_4^+. Table S1 summarizes the estimated values for 10 random angles (φ) measured between 0 and 2π.

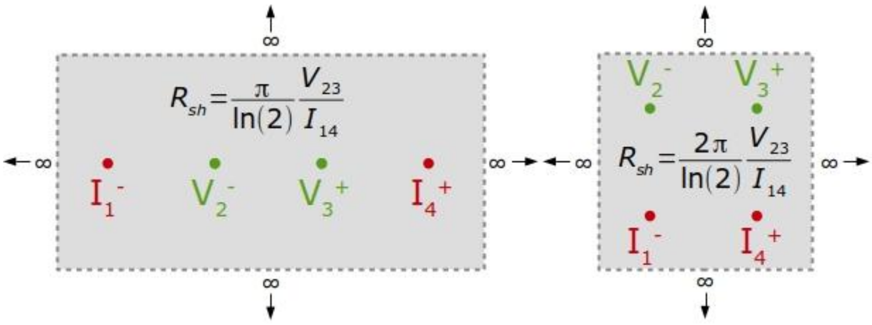

(a) **(b)**

Figure S13: Schematic view and equations for calculating the sheet resistance. a) Electrodes aligned equally spaced in a line and (b) electrodes located on the corners of a square with I^-, I^+ and V^-, V^+, current and voltage terminals, respectively.

Table S1: Electrical anisotropy measured for electrode geometries shown in Figure S13. a) A constant current of I = 1 µA was applied while the electrodes were randomly rotated sequentially increasing the angle φ between 0 and 2π. The voltage was measured and the resistance and sheet resistance were calculated.

position (#) 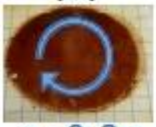 φ=0-2π	R_{4w}^{line} (Ω) 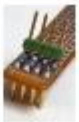	R_{4w}^{square} (Ω) 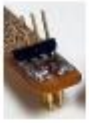	R_{sh}^{line} (Ω) 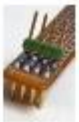	R_{sh}^{square} (Ω) 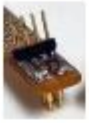
1	842	413	3816	3744
2	840	430	3807	3898
3	773	397	3503	3599
4	854	438	3871	3970
5	750	414	3399	3753
6	834	403	3780	3653
7	772	405	3499	3671
8	845	410	3830	3716
9	847	438	3839	3970
10	830	445	3762	4034
Average Value	**819**	**419**	**3711**	**3801**
Standard Deviation	**38**	**17**	**173**	**154**

S6.) Electro-thermal response - temperature dependence of voltage-current characteristics

Figure S14 – S16 show the voltage current characteristics measured within a homebuilt measurement chamber including temperature control showing values of applied current, values of measured voltage and linear fits of V-I curves done at different temperatures.

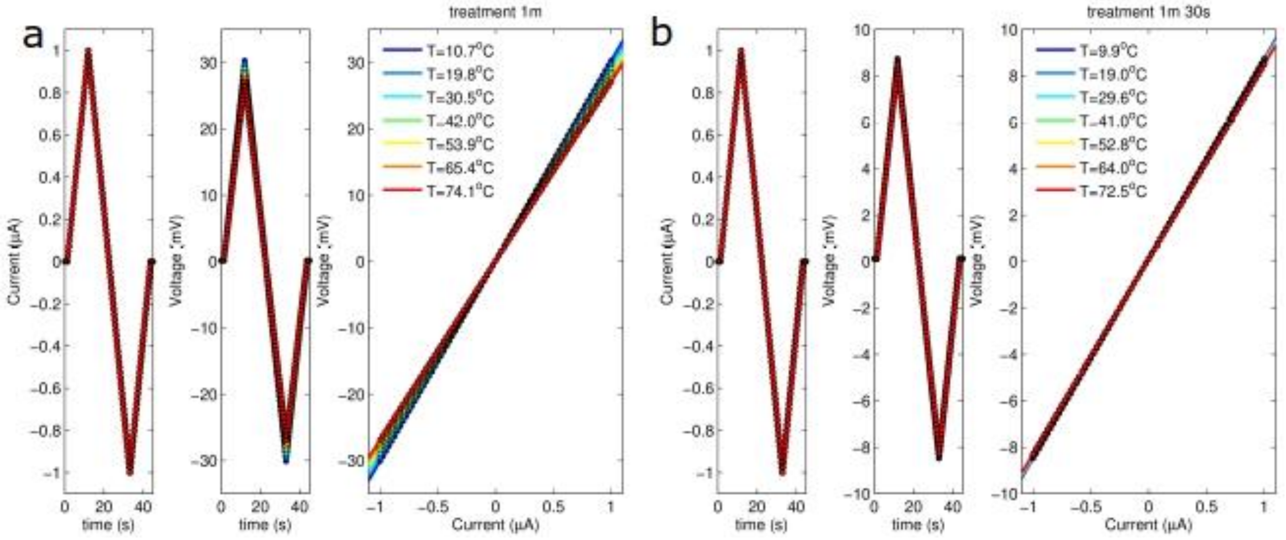

Figure S14: Voltage-Current characteristics upon heating. (a) Halogen vapor treatment time t = 60 seconds and (b) t = 90 seconds.

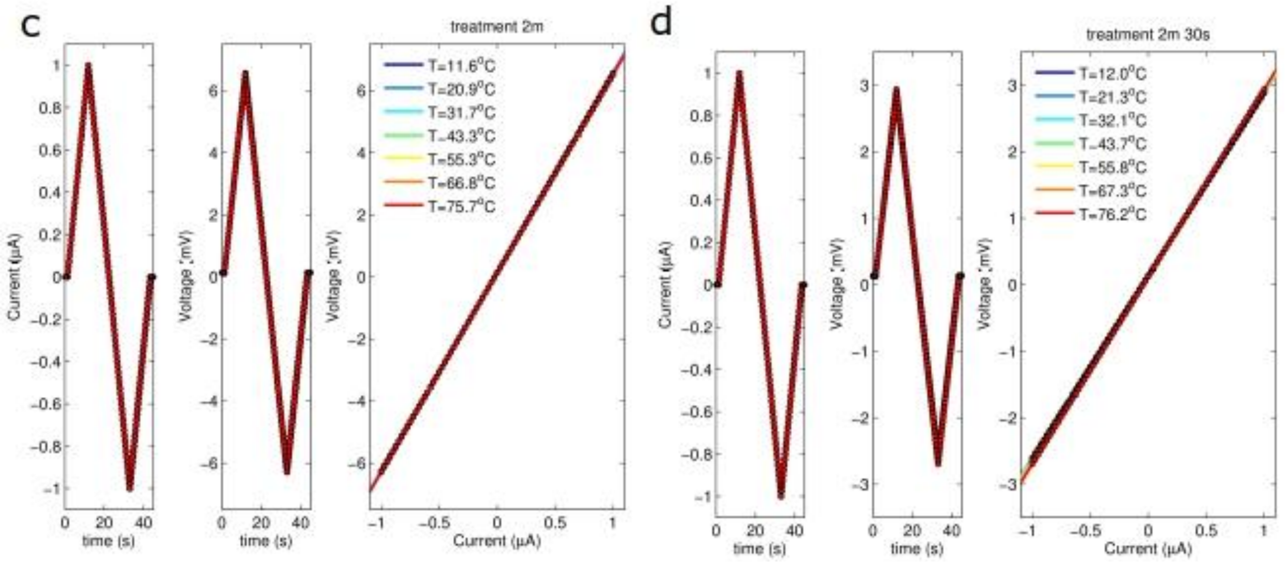

Figure S15: Voltage-Current characteristics upon heating. (c) Halogen vapor treatment time t = 120 seconds and (d) t = 150 seconds.

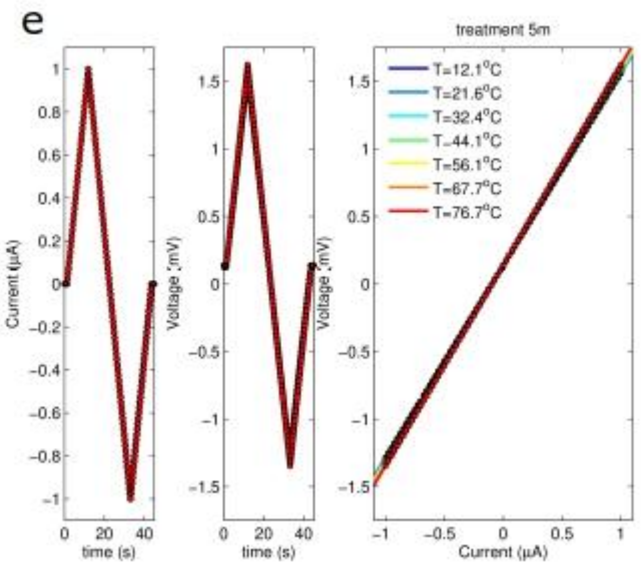

Figure S16: Voltage-Current characteristics upon heating. (e) Halogen vapor treatment time t = 300 seconds.

Equation EqS1 was used to fit the temperature response of all BL films and to extract first (ξ') and second (ξ") order temperature resistance coefficients:

$$R = R_{T0} \cdot [1 + \xi'(T - T_0) + \xi''(T - T_0)^2] \quad \text{(EqS1)}$$

Table S2: Electro-thermal response and extraction of first and second order temperature resistance coefficients. Sheet resistance (R_{sh}), first order temperature resistance coefficient (ξ'), second order temperature resistance coefficient (ξ") and quality of fit (R^2).

time (s)	R_{sh} (kΩ)	ξ′ (%/K)	ξ″ (%/K^2)	R^2
60	57.2	-0.18±0.02	(4.3±4.2)·10^{-4}	0.998
90	16.6	-0.057±0.008	(4.8±2.1)·10^{-4}	0.993
120	12.5	-0.024±0.001	(4.5±1.4)·10^{-4}	0.810
150	5.34	0.021±0.006	(6.3±1.3)·10^{-4}	0.998
300	2.14	0.06±0.01	(3.6±2.6)·10^{-4}	0.996

Arrhenius Equation, EqS2, was used to extract the activation energy for semiconductor-like films:

$$\sigma = \sigma_{25} \cdot e^{-\frac{E_a}{k_B T}} \tag{EqS2}$$

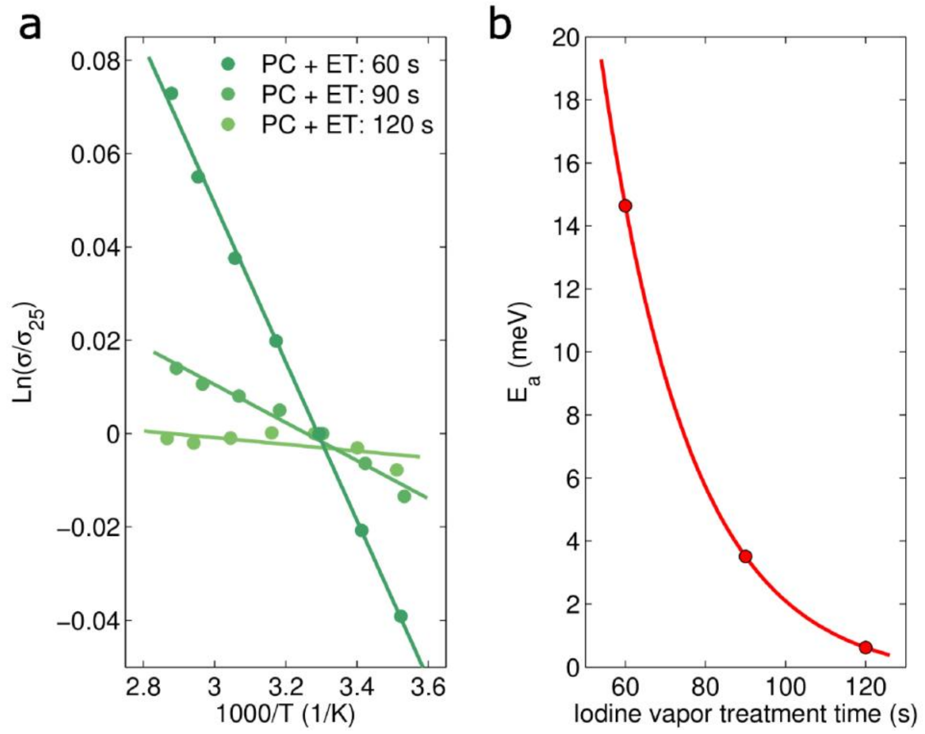

Figure S17: Electro-thermal response and extraction of activation energy (Ea). (a) Linear fits in Arrhenius plots and (b) activation energy (E_a) depending on halogen vapor treatment time.

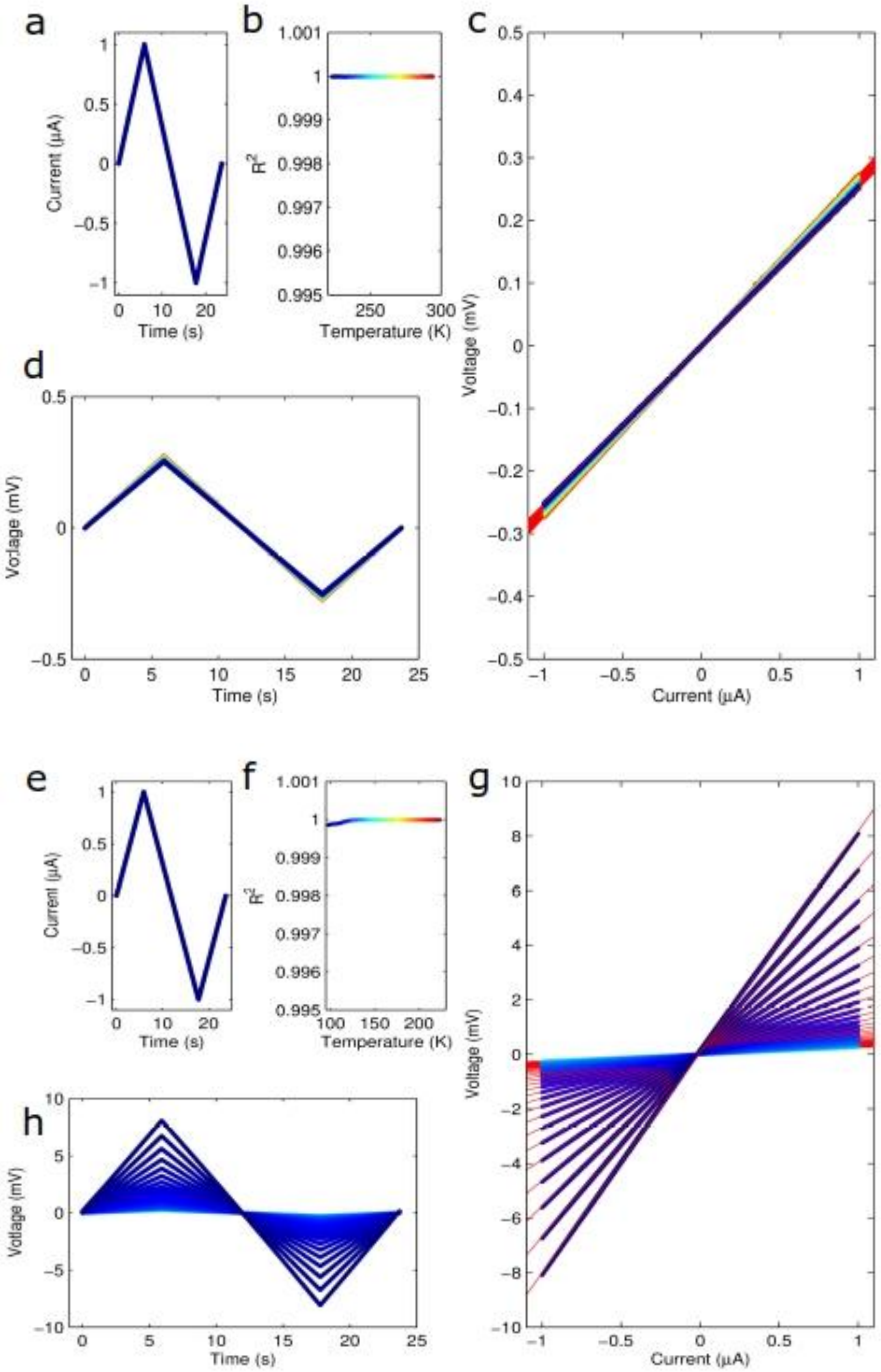

Figure S18: Low temperature Voltage-Current characteristics with corresponding linear fits and quality of fit (R^2). (a-d) Metal-like characteristics for temperatures between T = 220 K and T = 294 K; (e-h) insulator-like characteristics for temperatures between T = 95 K and T = 220 K.

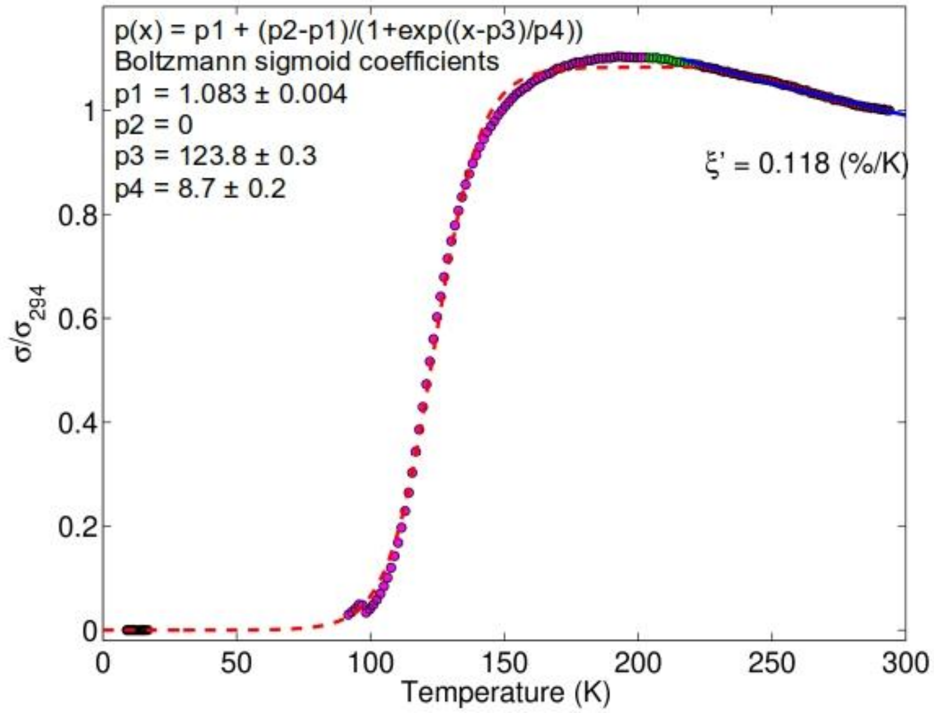

Figure S19: Low temperature relative conductivity (σ/σ_{294}). With Metal-like temperature dependence for temperatures from T = 220K – T = 294K (colored dots) and linear temperature resistance coefficient ξ' = 0.118 (%/K). Semiconductor-insulator transition for temperatures bellow T = 200 K. Dashed red line is best fit to Boltzmann sigmoid.

S7.) Optical response and extraction of bandgap and sub-bandgap states

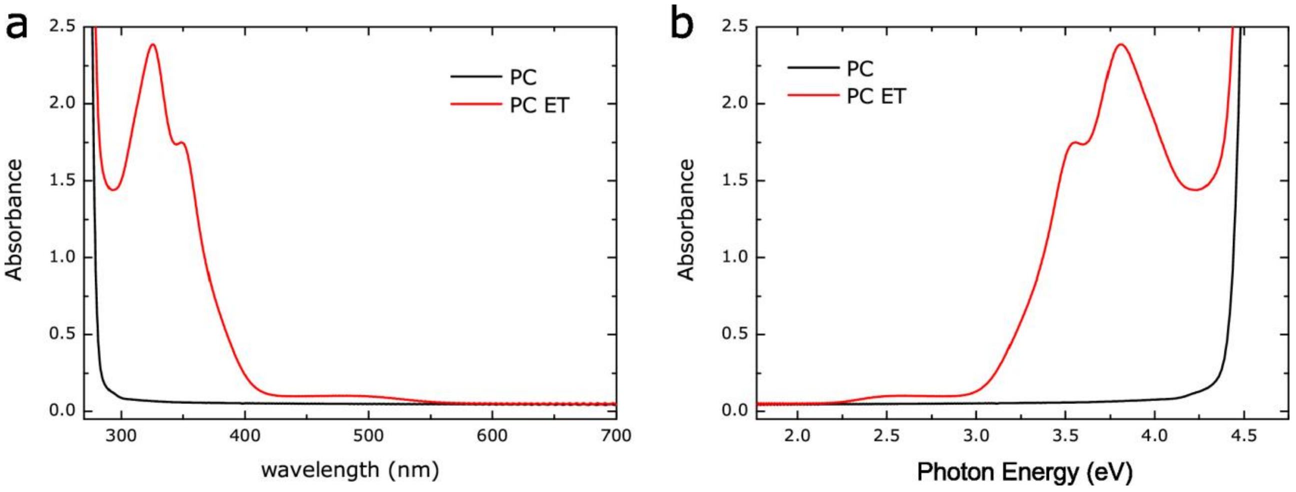

Figure S20: Optical absorption spectra for bare PC and PC with ET. (a) Absorbance as function of wavelength and (b) Absorbance as function of photon energy.

Tauc Equation, EqS3, was used to extract the bandgap:

$$\alpha(h\nu) \sim \frac{(h\nu - E_g)^2}{h\nu} \qquad \text{(EqS3)}$$

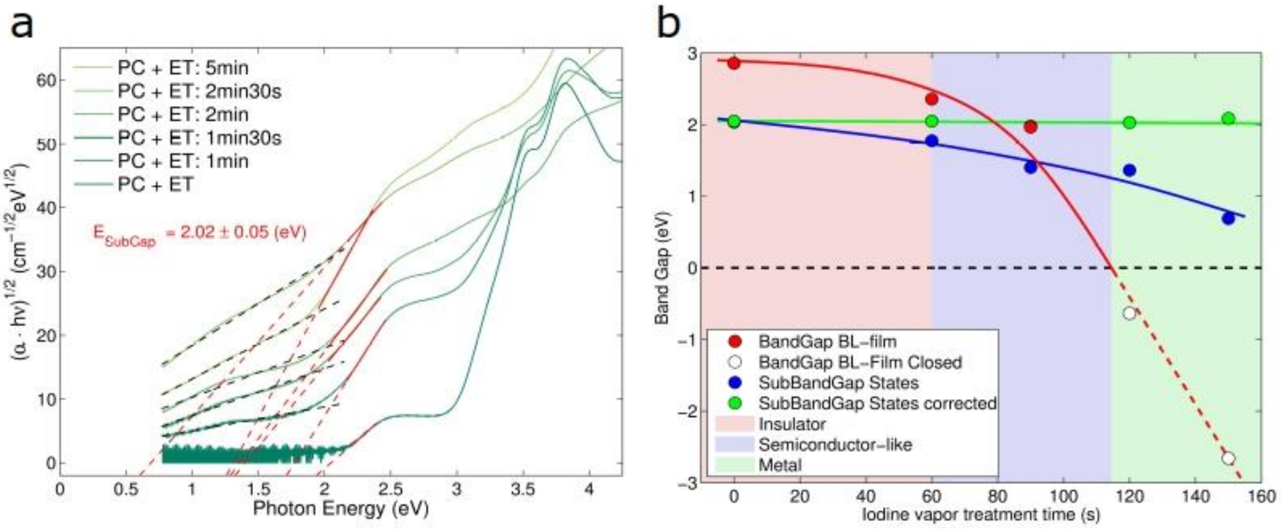

Figure S21: Optical response of solid-state films at different iodine treatment times. (a) Tauc plot with linear regression of sub bandgap states. (b) Comparison between states extracted in **Figure 4b** (red dots and line), sub bandgap states extracted in FigureS21a (blue dots and line, extracted as intercept of red broken line with x-axis), and corrected sub bandgap states (green dots and line, extracted as intercept between red and black dashed lines in Figure S21a).

S8.) Estimation of total film thickness by interference and micrometer gauge

Equation, EqS4, was used to estimate the total film thickness (d) with number of total maxima (or minima) between wavelengths λ_1 and λ_2, $\Theta = 90^o$ incident angle and n refractive index of polycarbonate $n = 1.60 \pm 0.04$. To compare the validity of the values extracted by interference, a micrometer gauge was used to measure the thickness mechanically. Results are summarized in Figure S23b.

$$d = \frac{\Delta m}{2\sqrt{n^2 - sin^2\Theta)}} \cdot \frac{1}{\frac{1}{\lambda_2} - \frac{1}{\lambda_1}} \quad \text{(EqS4)}$$

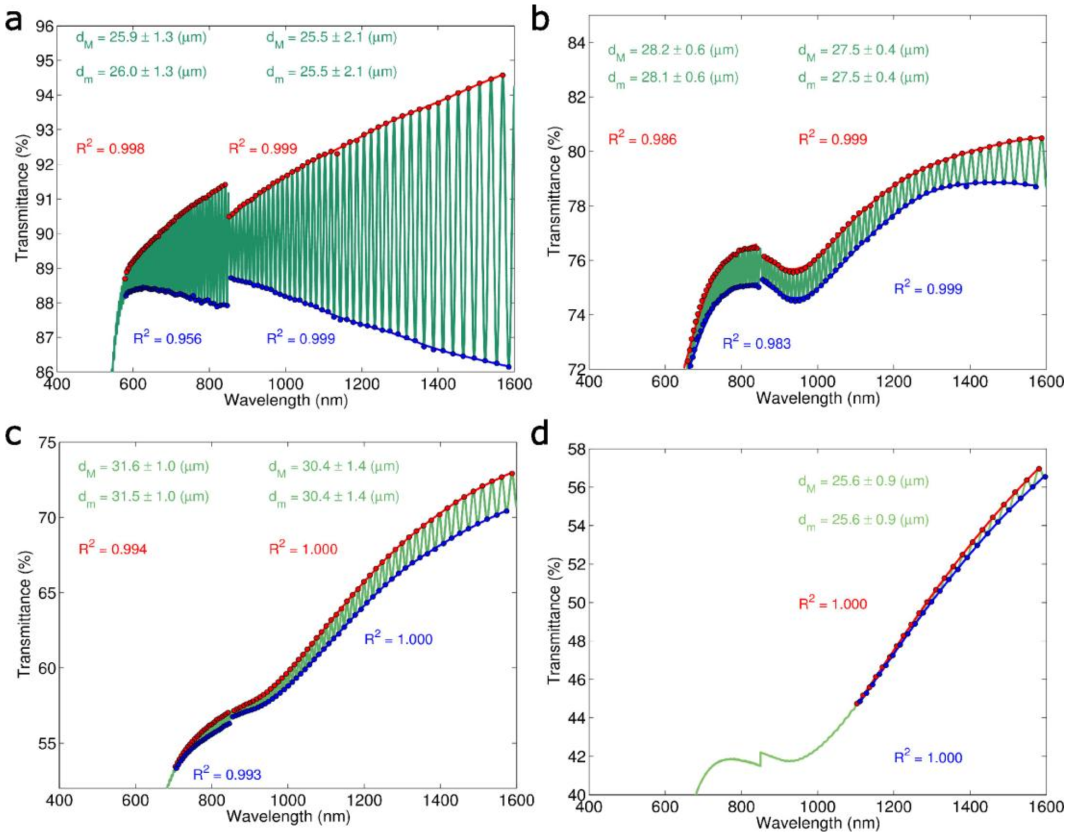

Figure S22: Estimation of total film thickness by interference. (a) PC + ET, (b) PC + ET treated with iodine for t = 60 s, (c) PC + ET treated with iodine for t = 90 s and (d) PC + ET treated with iodine for t = 120 s, respectively. Red fits correspond to maxima of transmittance (film thickness: d_M), blue fits correspond to minima of transmittance (film thickness: d_m). The notch at around 850 nm is an artifact of the UV-vis spectrometer. Film thickness was calculated before and after the notch (except for figure d, where the interference pattern appeared only at high wavelengths) using equation EqS4.

a

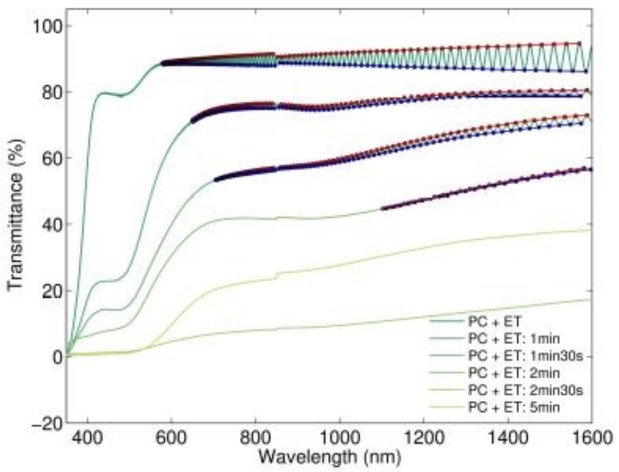

b

time (s)	Micrometer (um)	Interference (um)
0	27.2 ± 0.8	25.5 ± 2.1
60	30.6 ± 1.1	28.2 ± 0.6
90	30.0 ± 0.7	30.4 ± 1.4
120	26.6 ± 1.1	25.6 ± 0.9
150	29.6 ± 1.1	No interference
300	28.8 ± 1.5	No interference
	28.8 ± 1.6	29 ± 2

Figure S23: Estimation of total film thickness by interference. (a) Transmission spectra for films at different times of halogen treatment. Interference maxima are fitted with a red line, minima with a blue line, respectively. (b) Estimated thickness extracted by interference compared to thickness measured by micrometer gauge.

S9.) Prototype development: Pressure sensor and Temperature sensor

Prototype #1 comprises a BL-film exhibiting a low temperature resistance coefficient which was wrapped around a plastic tube. The tube was sealed on one side and connected to a pump on the other end. The pump allowed applying pressure values raging from 0 to 500 mbar in 10 steps in a reversible fashion. The pressure response of prototype #1 is shown in Figure S24 and exhibited a high sensitivity of 1.33 ± 0.03 Ω/mbar.

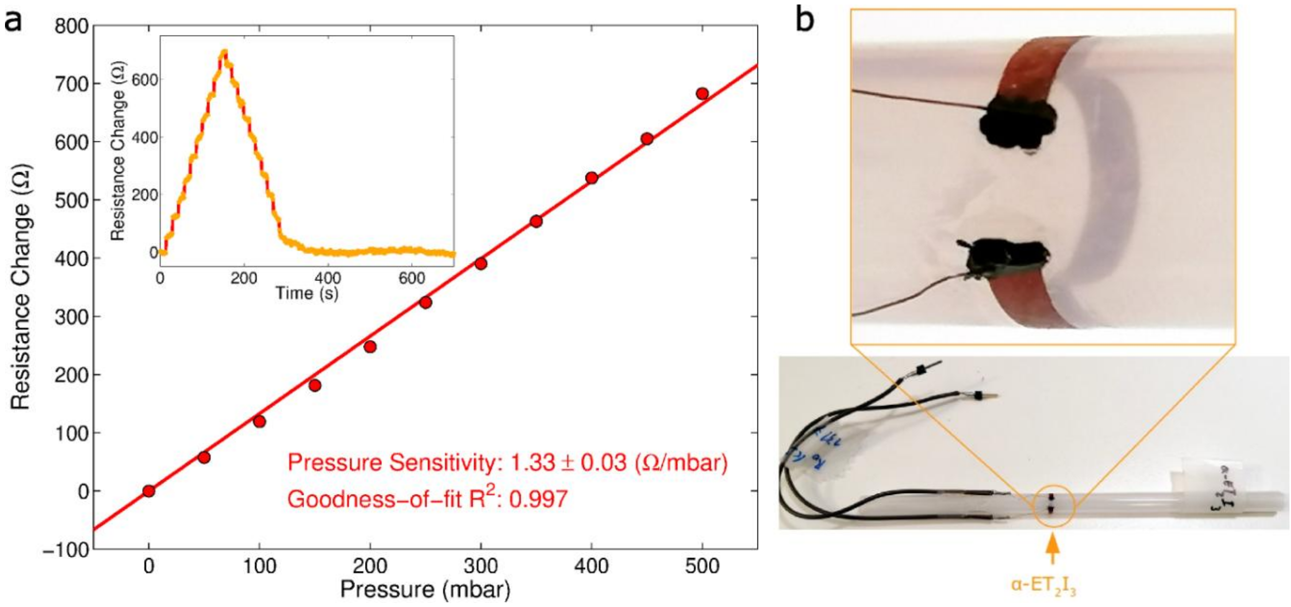

Figure S24: Prototype #1. (a) Resistance change of BL-film at different air pressure applied to the plastic tube, and (b) optical images of prototype #1.

For prototype #2 and #3 BL-films with both semiconductor-like (ξ' = -0.18 %/K) and metallic (ξ' = 0.12%/K) properties were surface mounted to the outside of a plastic tube. A platinum resistor (Pt_{1000}) was used as a reference sensor and mounted inside the tube in direct contact with the fluid (Figure 25b). To test the prototypes, water at different temperature was supplied at a flow rate of 2 ml/s. Figure S25 shows optical images of the prototype. Figure S26 and S27 show temperature response of a semiconductor-like and a metal-like sensor, respectively.

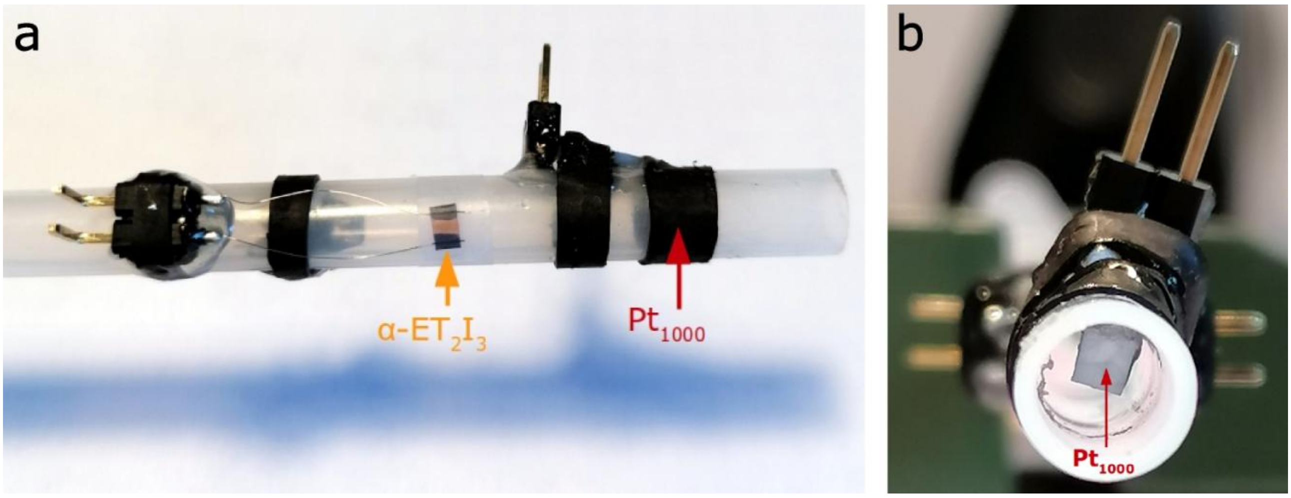

Figure S25: Prototype #2. (a) Optical image of prototype #2 with a semiconductor-like BL-film mounted on the tube surface. (b) Platinum reference sensor in direct contact with water flowing through the tube.

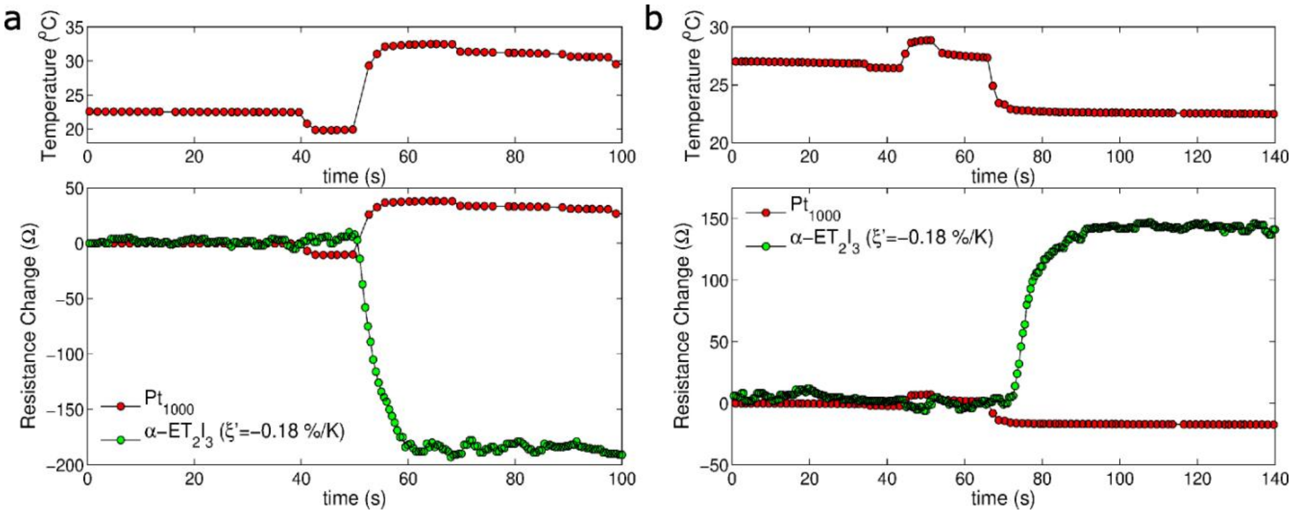

Figure S26: Prototype #2. Resistance change of BL-film at different temperature of the plastic tube with a clear semiconductor-like response; (a) heating, (b) cooling.

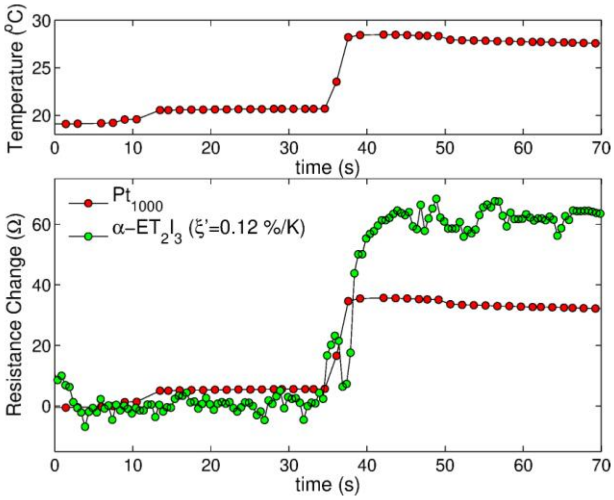

Figure S27: Prototype #3. Resistance change of BL-film at different temperature of the plastic tube with a clear metal-like response.

References:

1 Petsev, D. N., Chen, K., Gliko, O. & Vekilov, P. G. Diffusion-limited kinetics of the solution–solid phase transition of molecular substances. *Proceedings of the National Academy of Sciences* **100**, 792, (2003).

2 Laukhina, E., Pfattner, R., Ferreras Lourdes, R., Galli, S., Mas-Torrent, M., Masciocchi, N., Laukhin, V., Rovira, C. & Veciana, J. Ultrasensitive Piezoresistive All-Organic Flexible Thin Films. *Advanced Materials* **22**, 977-981, (2009).
